# Efficacy and safety of different acupuncture-related therapies for primary trigeminal neuralgia: a systematic review and network meta-analysis

**DOI:** 10.3389/fpain.2026.1854738

**Published:** 2026-07-14

**Authors:** Guanxi Ren, Yinsu Chen, Xinyu Zhang, Yaqing Guo, Weijia Peng, Kunping Jia, Jiawen Li, Yulong Shan, Xinyue Qiu, Guofeng Cai

**Affiliations:** 1 Graduate School of Heilongjiang University of Chinese Medicine, Harbin, China; 2 Department of Intensive Rehabilitation Medicine, The Second Affiliated Hospital of Heilongjiang University of Chinese Medicine, Harbin, China

**Keywords:** acupuncture, bloodletting therapy, network meta-analysis, pain, primary trigeminal neuralgia

## Abstract

**Background:**

Primary trigeminal neuralgia (PTN) is a debilitating neuropathic pain disorder with limited long-term treatment options. Although acupuncture is widely used, the comparative effectiveness of different acupuncture modalities remains unclear.

**Objective:**

To compare the efficacy and safety of various acupuncture therapies for PTN using a network meta-analysis (NMA).

**Methods:**

Randomized controlled trials were identified through a systematic search of seven databases from database inception to 2 March 2026. Outcomes included total effective rate (TER), visual analogue scale (VAS), attack frequency (AF), traditional Chinese medicine syndrome score (TCMSS), and adverse events (AEs). A network meta-analysis was conducted, and treatments were ranked using SUCRA.

**Results:**

This network meta-analysis included 58 randomized controlled trials. The results showed that bloodletting therapy was identified as a potentially effective intervention for improving TER (RR = 2.00, 95% CI: 1.15–3.47; SUCRA = 95.6%; CINeMA: low certainty) and reducing VAS scores (MD = 0.07, 95% CI: 0.01–0.61; SUCRA = 87.4%; CINeMA: very low certainty). In contrast, acupuncture combined with conventional Western medicine performed better in reducing AF (MD = 0.05, 95% CI: 0.02–0.14; SUCRA = 100%; CINeMA: very low certainty), improving TCMSS (SMD = 0.15, 95% CI: 0.07–0.34; SUCRA = 90.7%; CINeMA: very low certainty), and decreasing the risk of AEs (OR=0.18, 95% CI: 0.12–0.28; SUCRA = 73.8%; CINeMA: moderate certainty).

**Conclusions:**

Different acupuncture modalities may offer distinct therapeutic benefits for PTN. Bloodletting therapy showed potential advantages in improving TER and reducing VAS, whereas acupuncture combined with conventional Western medicine appeared to perform better in reducing AF, improving TCMSS, and lowering the risk of AEs. However, these findings should be interpreted cautiously because of moderate-to-high heterogeneity across some outcomes and the generally low certainty of the evidence. Further large-scale, multicenter, high-quality randomized controlled trials are warranted to confirm these findings.

**Systematic Review Registration:**

https://www.crd.york.ac.uk/prospero/, PROSPERO (CRD420261339021).

## 1 Introduction

Primary trigeminal neuralgia (PTN) is a common disorder of the cranial nerves. It is marked by repeated attacks of sudden, intense, electric shock–like or stabbing pain affecting one or more branches of the trigeminal nerve (1). The annual incidence is estimated at 4–28 cases per 100,000 people, increasing with age and occurring slightly more often in women than in men. PTN substantially impairs quality of life and negatively affects psychological well-being (2). The leading explanation for its cause is neurovascular compression. In this model, a nearby blood vessel repeatedly presses on the trigeminal nerve near its entry into the brainstem, leading to local damage of the nerve's protective covering. This damage disrupts normal signal transmission, causing inappropriate activation of nerve fibers. Over time, ongoing pain signals may also increase the sensitivity of the central nervous system, further intensifying pain and promoting spontaneous episodes (3, 4).

Current management of PTN relies mainly on medications and surgery, both of which have important drawbacks. Guidelines from the European Academy of Neurology recommend carbamazepine (CBZ) and oxcarbazepine (OXC) as first-line treatments (5). However, prolonged use is often limited by side effects such as dizziness and impaired coordination, and in some cases by liver or kidney dysfunction. Over time, some patients also develop reduced responsiveness to these drugs, leading to diminished benefit (6). When medications are ineffective or poorly tolerated, surgical approaches–such as microvascular decompression, radiofrequency ablation, and Gamma Knife radiosurgery–may be considered. Among these, microvascular decompression is the only treatment that addresses the underlying cause. However, it requires open cranial surgery and carries risks including cerebrospinal fluid leakage, hearing loss, and, in rare cases, brainstem injury. Recurrence after surgery also remains a concern (7). In light of these challenges, there is an increasing need to identify alternative or adjunctive treatments that are both effective and well tolerated, with the potential to improve long-term patient outcomes.

Acupuncture, a core modality within traditional Chinese medicine and complementary therapies, shows considerable promise for the management of PTN. Clinical studies indicate that it not only relieves pain but also improves psychological well-being—reducing anxiety and depression—and enhances overall quality of life. Importantly, acupuncture is generally well tolerated and associated with a low rate of serious adverse events (8). Its therapeutic effects appear to arise from multiple, complementary mechanisms. Recent systematic reviews indexed in major databases, including PubMed, have increasingly supported the effectiveness of acupuncture for PTN. Evidence suggests that acupuncture can significantly reduce pain intensity and attack frequency in affected patients. For example, a systematic review by Hu reported that, compared with carbamazepine alone, acupuncture achieved better clinical outcomes, including higher total effective rate (TER) and greater reductions in visual analogue scale (VAS) scores, while also resulting in fewer adverse events (9). Acupuncture promotes the release of endogenous opioids in the central nervous system, such as endorphins and enkephalins, while reducing levels of pain-related neuropeptides, including substance P and calcitonin gene-related peptide, thereby limiting inflammatory responses linked to pain. In parallel, functional magnetic resonance imaging studies suggest that acupuncture can modulate brain networks involved in pain processing, which may help reduce heightened pain sensitivity (10, 11).

Although the number of randomized controlled trials (RCTs) and conventional meta-analyses on acupuncture for PTN has grown in recent years, important gaps in the evidence remain. Acupuncture includes a range of distinct approaches, such as manual acupuncture, electroacupuncture, warming needle moxibustion, bloodletting therapy, and acupoint injection. However, most studies focus on comparing a single acupuncture method with standard Western medication, or they combine different acupuncture approaches into one group. These strategies may obscure meaningful differences in effectiveness between individual treatments (8). As a result, robust evidence directly comparing the efficacy and safety of different acupuncture modalities for PTN is still lacking. This limits the ability to identify the most effective treatment and leaves clinicians without clear guidance for decision-making.

This study will use a network meta-analysis (NMA) to combine direct and indirect evidence, allowing a comprehensive comparison of the efficacy and safety of commonly used acupuncture therapies for PTN. In addition, probabilistic ranking will be applied to identify the relative performance of each intervention. By addressing current gaps in the evidence, this study seeks to provide more reliable data to support clinical decision-making and guide the selection of optimal acupuncture treatment strategies for PTN.

## 2 Methods

### 2.1 Literature search strategy

This NMA followed the PRISMA extension for systematic reviews, with specific consideration of guidance for network meta-analyses of healthcare interventions (12, 13). The PRISMA checklist is presented in Supplementary Appendix S1. The study protocol was prospectively registered in the International Prospective Register of Systematic Reviews (PROSPERO; CRD420261339021).

A comprehensive search was conducted across major electronic databases, including PubMed, Web of Science, Embase, and the Cochrane Library. To ensure broad coverage, key Chinese databases—China National Knowledge Infrastructure (CNKI), VIP Database, and WanFang Data—were also searched. The search spanned from database inception to March 2, 2026. Search terms were defined in both English and Chinese. The English search included terms such as “primary trigeminal neuralgia,” “acupuncture,” “moxibustion,” “electroacupuncture,” “acupuncture with mild moxibustion,” and “randomized controlled trial.” The search strategy was structured using the PICOS framework, combining subject headings with free-text terms to ensure comprehensive retrieval. Detailed search strategies for each database are provided in Supplementary Appendix S2.

### 2.2 Inclusion criteria

Eligible studies enrolled patients with a confirmed diagnosis of PTN. The diagnosis of primary trigeminal neuralgia (PTN) was based on the criteria for “classical trigeminal neuralgia” outlined in the 2018 *International Classification of Headache Disorders, 3rd edition* (ICHD-3) published by the International Headache Society Classification Committee (14). Diagnosis required recurrent unilateral facial pain occurring in at least three attacks and confined to one or more branches of the trigeminal nerve, without radiation beyond the trigeminal distribution. The pain had to exhibit at least three of the following features: paroxysmal attacks lasting from a fraction of a second to 2 min, severe intensity, an electric shock–like or stabbing quality, and provocation by innocuous stimuli such as light touch. In addition, patients showed no clinically significant neurological deficits apart from possible neurovascular compression, and the symptoms could not be better explained by another diagnosis within the ICHD-3 classification. No restrictions were placed on gender, ethnicity, or other demographic characteristics. Only RCTs were included. In the intervention arms, patients received acupuncture-related therapies, such as electroacupuncture, moxibustion, acupoint injection, acupoint embedding, intradermal acupuncture, or bloodletting therapy. The control group received standard Western pharmacological treatment, including CBZ and gabapentin. Studies evaluating these therapies alone or in combination with conventional Western medicine were considered eligible. The predefined outcomes included total effective rate (TER), Visual Analogue Scale (VAS) score, attack frequency (AF), Traditional Chinese Medicine Syndrome Score (TCMSS), and adverse events (AEs). TER was the primary dichotomous outcome and was extracted according to the efficacy criteria reported in the original studies. In most trials, treatment response was classified as cured, markedly effective, effective, or ineffective based on improvements in pain intensity, attack frequency and duration, and overall clinical symptoms. For the present analysis, TER was defined as the proportion of patients categorized as cured, markedly effective, or effective. Pain intensity was assessed using the VAS, with lower scores indicating less severe pain. AF referred to the number of trigeminal neuralgia episodes occurring within a specified period, as defined in each study. TCMSS was evaluated according to changes in traditional Chinese medicine syndrome scores, with lower scores indicating greater symptom improvement. Safety was assessed based on the incidence of AEs, including acupuncture-related discomfort and other treatment-related complications reported in the original studies. When multiple follow-up time points were reported, data collected immediately after treatment completion were preferentially extracted to improve comparability across studies. To reduce heterogeneity related to differences in outcome definitions, two reviewers independently evaluated and standardized the assessment criteria during data extraction. Because no universally accepted criteria exist for evaluating acupuncture efficacy in primary trigeminal neuralgia, outcome measures were based on the definitions used in the original studies and harmonized where possible during data synthesis.

### 2.3 Exclusion criteria

The exclusion criteria were defined as follows. Review articles, systematic reviews, animal studies, expert opinions, case reports, and other non-original clinical studies were excluded. Studies involving patients with secondary trigeminal neuralgia or unclear diagnostic criteria were also excluded. Studies were further excluded if the interventions were unrelated to the study objective or if complex combined therapies prevented the extraction of valid outcome data. In addition, studies with low methodological quality, incomplete outcome reporting, or unreliable data were excluded if these issues prevented valid data extraction or quantitative synthesis. This included inconsistencies in reported outcomes, discrepancies between statistical results and original data, duplicate publications, and missing key information such as sample size, means, standard deviations, or event numbers that could not be obtained after contacting the authors. Studies without accessible full texts were also excluded. Finally, studies with substantial baseline imbalances between groups and inadequate comparability were excluded.

### 2.4 Study selection and data extraction

Two reviewers independently screened the literature using EndNote in a structured, stepwise process. After removing duplicates, titles and abstracts were reviewed to exclude clearly irrelevant studies. The full texts of potentially eligible articles were then assessed to determine final inclusion. Disagreements were resolved through discussion, with a third reviewer consulted when necessary to reach consensus. Once the studies were selected, key data were systematically extracted. These included the first author's name, year of publication, sample size, and baseline characteristics of participants (such as age and gender). Information on random sequence generation, details of the interventions, and outcome measures used to assess study results was also collected.

### 2.5 Quality assessment of included studies and grading of the evidence

This study employed a rigorous, blinded approach to quality assessment. Two independent reviewers evaluated the methodological quality of the included studies using the Risk of Bias 2.0 tool recommended in the *Cochrane Handbook for Systematic Reviews of Interventions* (15). Any disagreements were resolved by consulting a third reviewer, with final decisions reached through structured discussion to ensure consistency and consensus. The quality of evidence was systematically assessed using the Confidence in Network Meta-Analysis (CINeMA) framework. It is specifically designed to evaluate the certainty of evidence in network meta-analyses. This method evaluated the reliability of the findings across six domains: within-study bias, reporting bias, indirectness, imprecision, heterogeneity, and incoherence.

### 2.6 Statistical analysis

NMA was performed using Stata version 18.0. Dichotomous outcomes were expressed as odds ratios (OR) or risk ratios (RR), while continuous outcomes were summarized as mean differences (MD) or standardized mean differences (SMD). All results are presented with 95% confidence intervals (95% CI). When closed loops were present in the evidence network, inconsistency between direct and indirect comparisons was assessed using the node-splitting method. A consistency model was applied if no significant inconsistency was detected (*P* > 0.05); otherwise, an inconsistency model was used. Heterogeneity was evaluated using the *τ*^2^ and classified as low (<0.04), low to moderate (0.04–0.16), moderate to high (0.16–0.36), or high (>0.36), in line with established guidelines (16, 17). For continuous outcomes, change scores were calculated as baseline values minus post-treatment values. Accordingly, larger positive change scores indicate greater symptom improvement. Therefore, a positive MD reflects greater improvement in the intervention group than in the control group, whereas a negative MD indicates greater improvement in the control group. Pairwise comparisons between interventions were summarized in league tables. To further compare treatments, the surface under the cumulative ranking curve (SUCRA) was calculated, with higher values indicating greater efficacy and safety. Potential publication bias and small-study effects were assessed using funnel plots.

## 3 Results

### 3.1 Literature search

**Figure 1:**
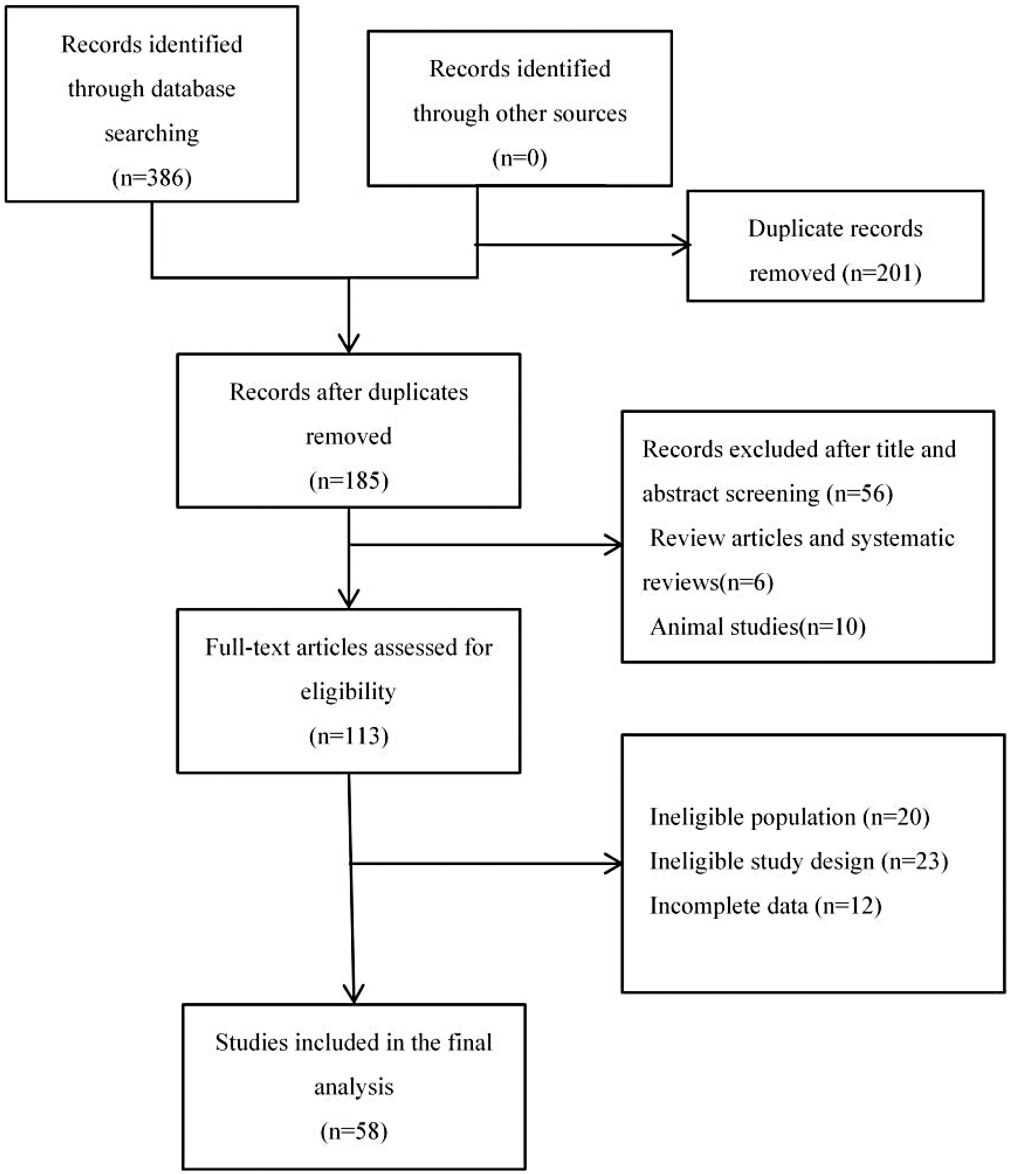
PRISMA flow diagram of study selection.

A total of 386 potentially relevant studies were identified through database searches, and no additional records were obtained from other sources (*n* = 0). After removing 201 duplicate records, 185 studies remained for screening. Title and abstract screening excluded studies that did not meet the inclusion criteria (*n* = 56), along with reviews or systematic reviews (*n* = 6) and animal studies (*n* = 10). The remaining 113 full-text articles were assessed for eligibility. Studies were further excluded because of ineligible study populations (*n* = 20), inappropriate study designs (*n* = 23), and incomplete data reporting (*n* = 12). Ultimately, 58 studies were included in the quantitative analysis. The study selection process is presented in Figure 1.

### 3.2 Characteristics of included studies

A total of 58 RCTs (18–75) published from database inception to 2 March 2026 were included in this analysis. All were two-arm studies and evaluated 10 intervention strategies: acupuncture, moxibustion, bloodletting therapy, electroacupuncture, acupoint embedding therapy, acupuncture combined with conventional Western medicine, electroacupuncture combined with conventional Western medicine, acupoint injection combined with conventional Western medicine, intradermal needle therapy combined with conventional Western medicine, and moxibustion combined with conventional Western medicine. Across all studies, participants were patients with PTN. The mean age ranged from 40 to 60 years, and treatment duration varied from 14 to 40 days. Outcome measures were broadly consistent, including TER, pain intensity assessed by the VAS, AF, TCMSS, and AEs. Further details of the included studies are presented in Supplementary Appendix S3 and S4.

### 3.3 Quality assessment

The risk of bias in all 58 RCTs was evaluated using the Cochrane RoB 2.0 tool (Supplementary Appendix S5, Figure S5, Table S5). Most studies demonstrated adequate randomization, with 53 trials (91.3%) rated as low risk and 5 (8.7%) as high risk, although some lacked sufficient methodological detail. Concerns were more evident in the implementation of interventions. A total of 56 trials (96.6%) were rated as having “some concerns,” with only 1 trial each classified as low risk and high risk. For outcome measurement, 53 trials (91.3%) were judged to be at low risk, while 5 (8.7%) were at high risk, likely reflecting limited use of blinding or non-standardized assessment methods. In contrast, all studies were considered low risk for both selective reporting and missing outcome data. Overall, 52 trials (89.6%) were assessed as having “some concerns,” 5 (8.7%) as high risk, and 1 (1.7%) as low risk. These limitations were mainly due to incomplete reporting of randomization procedures, lack of blinding, and absence of trial registration. Such features are common in open-label traditional Chinese medicine trials conducted in Chinese clinical settings and should be considered when interpreting the findings of this network meta-analysis.Heterogeneity varied across outcomes. The *τ*^2^ estimates indicated low heterogeneity for TER and AEs across the network (*τ*^2^ < 0.04). In contrast, moderate to substantial heterogeneity was observed for the visual analogue scale (VAS; *τ*^2^ = 0.81), Traditional Chinese Medicine Syndrome Scoring (TCMSS; *τ*^2^ = 1.06), and attack frequency (AF; *τ*^2^ = 1.56), suggesting greater variability in study outcomes for these measures. Funnel plots showed no clear asymmetry, suggesting a low likelihood of publication bias (Supplementary Appendix 10). Using the CINeMA framework, the certainty of evidence for most comparisons was rated as low to moderate, with none reaching a high level of confidence (Supplementary Appendix S9). All networks met the assumption of transitivity, supporting the validity of indirect comparisons (Supplementary Appendix S9, Table S9.1). Sensitivity analyses were conducted to assess the robustness of the findings. Studies at high risk of bias and those involving complex combination therapies were excluded to reduce the potential influence of methodological limitations and clinical heterogeneity. The results remained largely consistent with those of the primary analysis across the main outcomes, supporting the stability and reliability of the findings (Supplementary Appendix S11). In addition, treatment duration was used as a covariate in a meta-regression analysis to examine its potential effect on the outcomes. The results showed no significant association between treatment duration and the estimated effects, and the overall conclusions were consistent with those of the primary analysis (Supplementary Appendix S12). However, given the moderate-to-high heterogeneity observed in some outcomes and the generally limited certainty of the evidence, these findings should be interpreted with caution.

### 3.4 Results analysis

#### 3.4.1 TER

**Figure 2:**
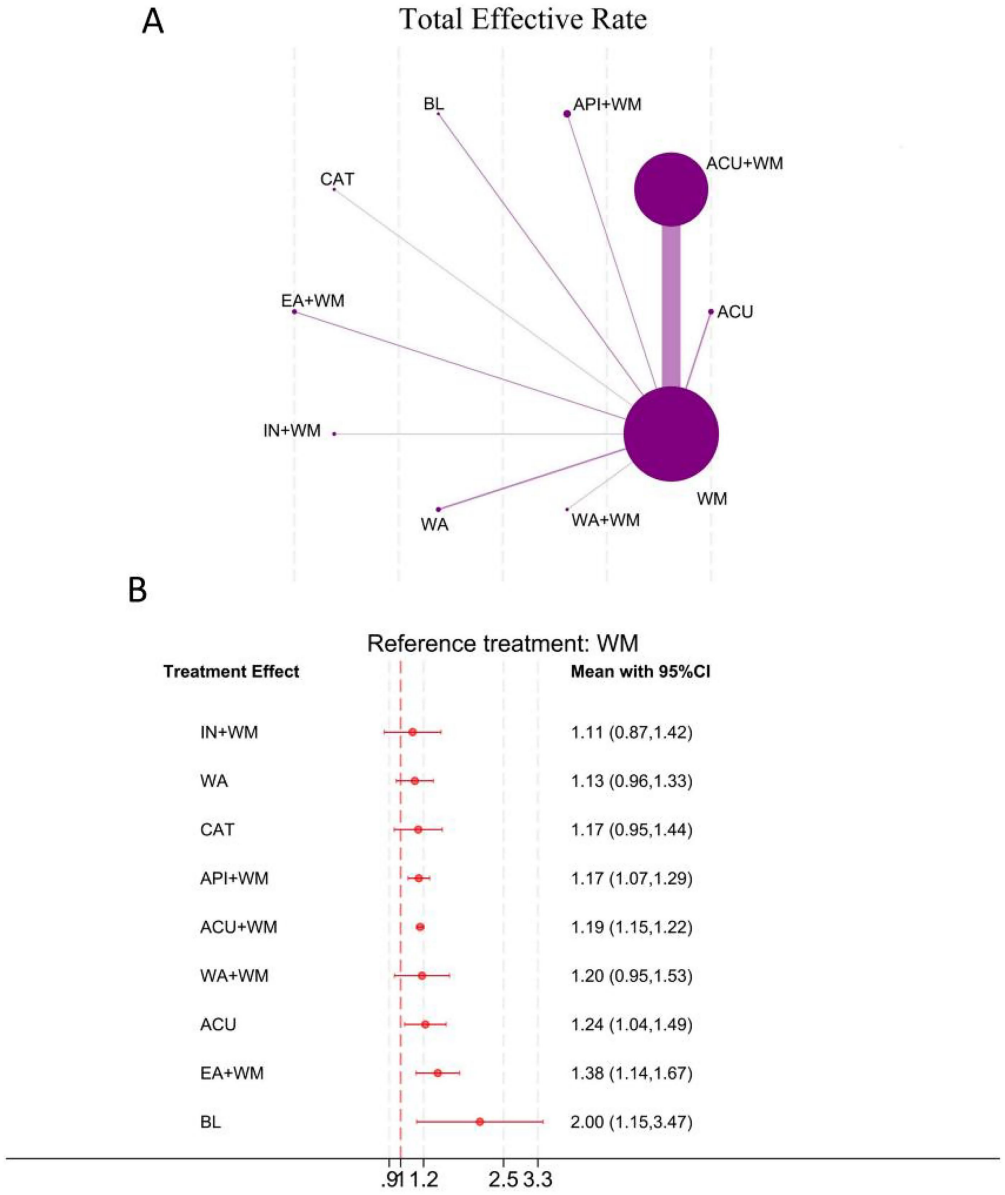
Network map of the effect on TER, and forest plot of network effect sizes compared with WM. **(A)** Network map of the effect on TER. **(B)** Forest plot of network effect sizes compared with WM.

For TER, this network meta-analysis included 56 RCTs, covering 10 interventions and a total of 4,036 participants. The evidence network (Figure 2A) had a star-shaped configuration, with conventional Western medicine acting as the central comparator and directly linked to all nine acupuncture-related therapies. As shown in Figure 2B, five interventions were associated with significantly better outcomes. Bloodletting therapy demonstrated the largest effect (RR = 2.00, 95% CI: 1.15–3.47; SUCRA = 95.6%; CINeMA: low certainty). This was followed by electroacupuncture combined with conventional Western medicine (RR = 1.38, 95% CI: 1.14–1.67; SUCRA = 81.3%; CINeMA: moderate certainty), acupuncture alone (RR = 1.24, 95% CI: 1.04–1.49; SUCRA = 60.2%; CINeMA: low certainty), acupuncture combined with conventional Western medicine (RR = 1.19, 95% CI: 1.15–1.22; SUCRA = 48.9%; CINeMA: moderate certainty), and acupoint injection combined with conventional Western medicine (RR = 1.17, 95% CI: 1.07–1.29; SUCRA = 44.7%; CINeMA: moderate certainty). The league table indicated no statistically significant differences between any pairwise comparisons of the interventions. Detailed comparative results for TER are presented in the SUCRA rankings (Supplementary Appendix S7, Figure S7.1, Table S7.1) and the league table (Supplementary Appendix S8, Table S8.1).

#### 3.4.2 VAS

**Figure 3:**
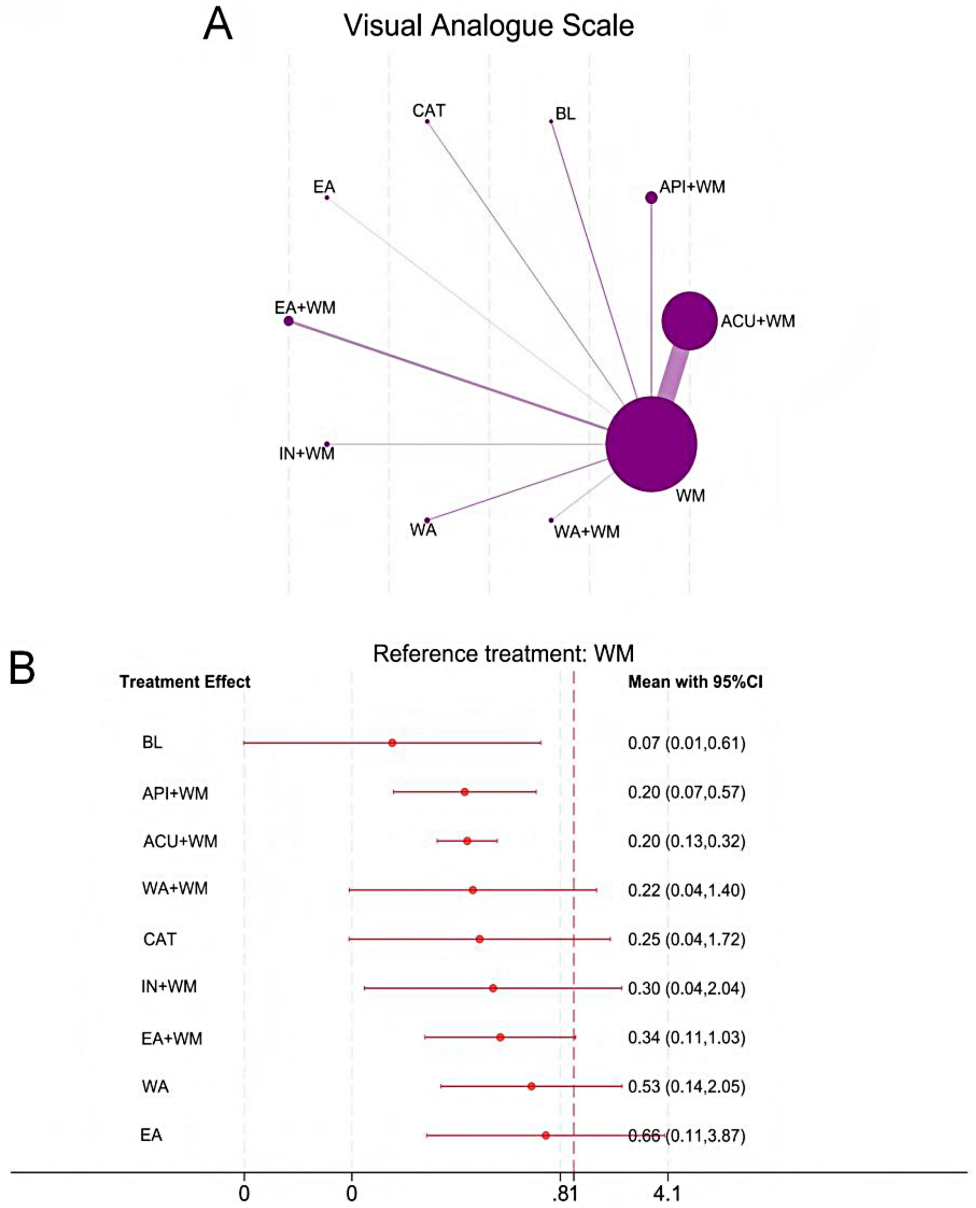
Network map of the effect on VAS, and forest plot of network effect sizes compared with WM. **(A)** Network map of the effect on VAS. **(B)** Forest plot of network effect sizes compared with WM.

For the VAS, this network meta-analysis included 32 RCTs, covering 10 interventions and a total of 2,228 participants. The evidence network (Figure 3A) had a star-shaped structure, with conventional Western medicine at its center, directly linked to all nine acupuncture-related therapies. As shown in Figure 3B, three interventions were significantly more effective. Bloodletting therapy showed the greatest reduction in VAS scores (MD = 0.07, 95% CI: 0.01–0.61; SUCRA = 87.4%; CINeMA: very low certainty). Acupoint injection combined with conventional Western medicine (MD = 0.20, 95% CI: 0.07–0.57; SUCRA = 66.5%; CINeMA: very low certainty) and acupuncture combined with conventional Western medicine (MD = 0.20, 95% CI: 0.13–0.32; SUCRA = 66.5%; CINeMA: very low certainty) also showed significant benefits. The league table indicated no statistically significant differences between any pairwise comparisons of the interventions. Further details are provided in the SUCRA ranking plots (Supplementary Appendix S7, Figure S7.2, Table S7.2) and the league tables (Supplementary Appendix S8, Table S8.2).

#### 3.4.3 AF

**Figure 4:**
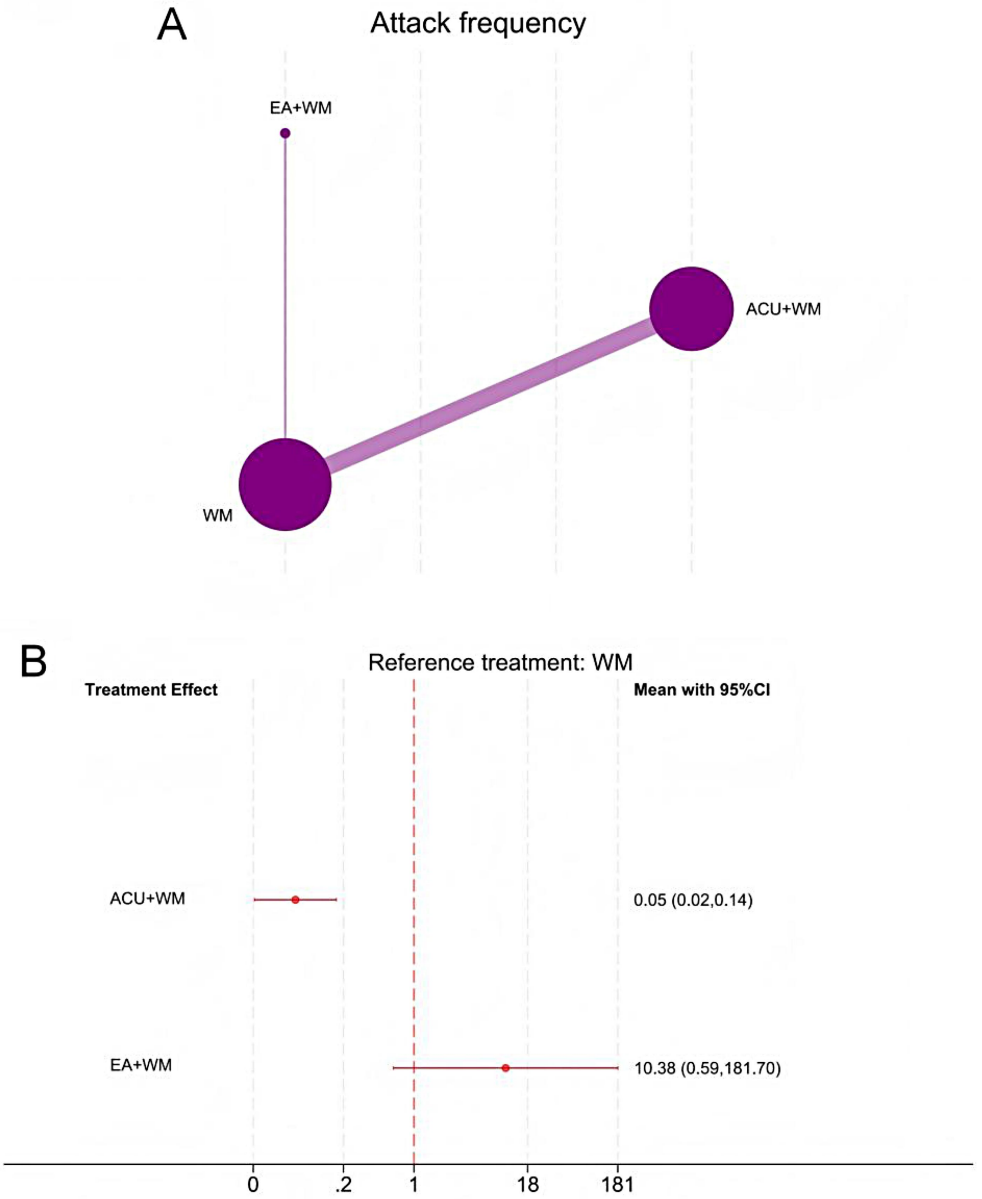
Network map of the effect on AF, and forest plot of network effect sizes compared with WM. **(A)** Network map of the effect on AF. **(B)** Forest plot of network effect sizes compared with WM.

For AF, this network meta-analysis included nine RCTs, comprising three intervention nodes and a total of 689 participants. The evidence network (Figure 4A) formed a star-shaped structure, with conventional Western medicine as the central comparator, directly linked to both acupuncture-related interventions. As shown in Figure 4B, only one intervention demonstrated a statistically significant advantage. Acupuncture combined with conventional Western medicine was associated with a significant reduction in attack frequency (MD = 0.05, 95% CI: 0.02–0.14; SUCRA = 100%; CINeMA: very low certainty). The league table showed that acupuncture combined with conventional Western medicine was more effective than electroacupuncture combined with conventional Western medicine in reducing AF. Further details on comparative effects are provided in the SUCRA rankings (Supplementary Appendix S7, Figure S7.3, Table S7.3) and the league table (Supplementary Appendix S8, Table S8.3).

#### 3.4.4 TCMSS

**Figure 5:**
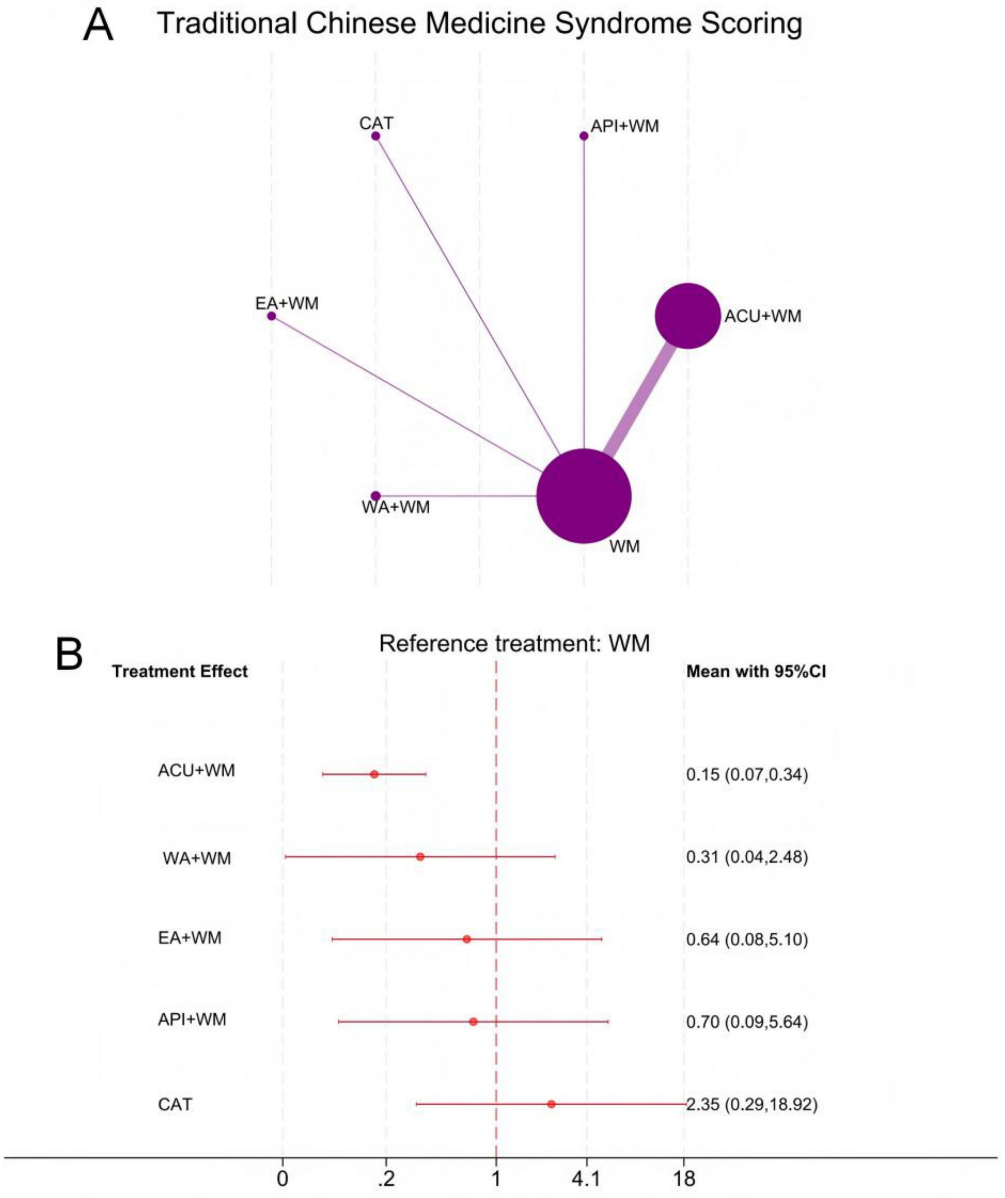
Network map of the effect on TCMSS, and forest plot of network effect sizes compared with WM. **(A)** Network map of the effect on TCMSS. **(B)** Forest plot of network effect sizes compared with WM.

For the TCMSS, this network meta-analysis included 11 RCTs, covering six interventions and a total of 875 participants. The evidence network (Figure 5A) formed a star-shaped structure, with conventional Western medicine at the center, directly linked to all five acupuncture-based therapies. As illustrated in Figure 5B, only one intervention showed a statistically significant advantage. Acupuncture combined with conventional Western medicine showed the greatest benefit (SMD = 0.15, 95% CI: 0.07–0.34; SUCRA = 90.7%; CINeMA: very low certainty). The league table showed that acupuncture combined with conventional Western medicine was more effective than acupoint injection combined with conventional Western medicine in reducing TCMSS. Detailed comparative results for TCMSS are provided in the SUCRA ranking plots (Supplementary Appendix S7, Figure S7.4, Table S7.4) and the league table (Supplementary Appendix S8, Table S8.4).

#### 3.4.5 AEs

**Figure 6:**
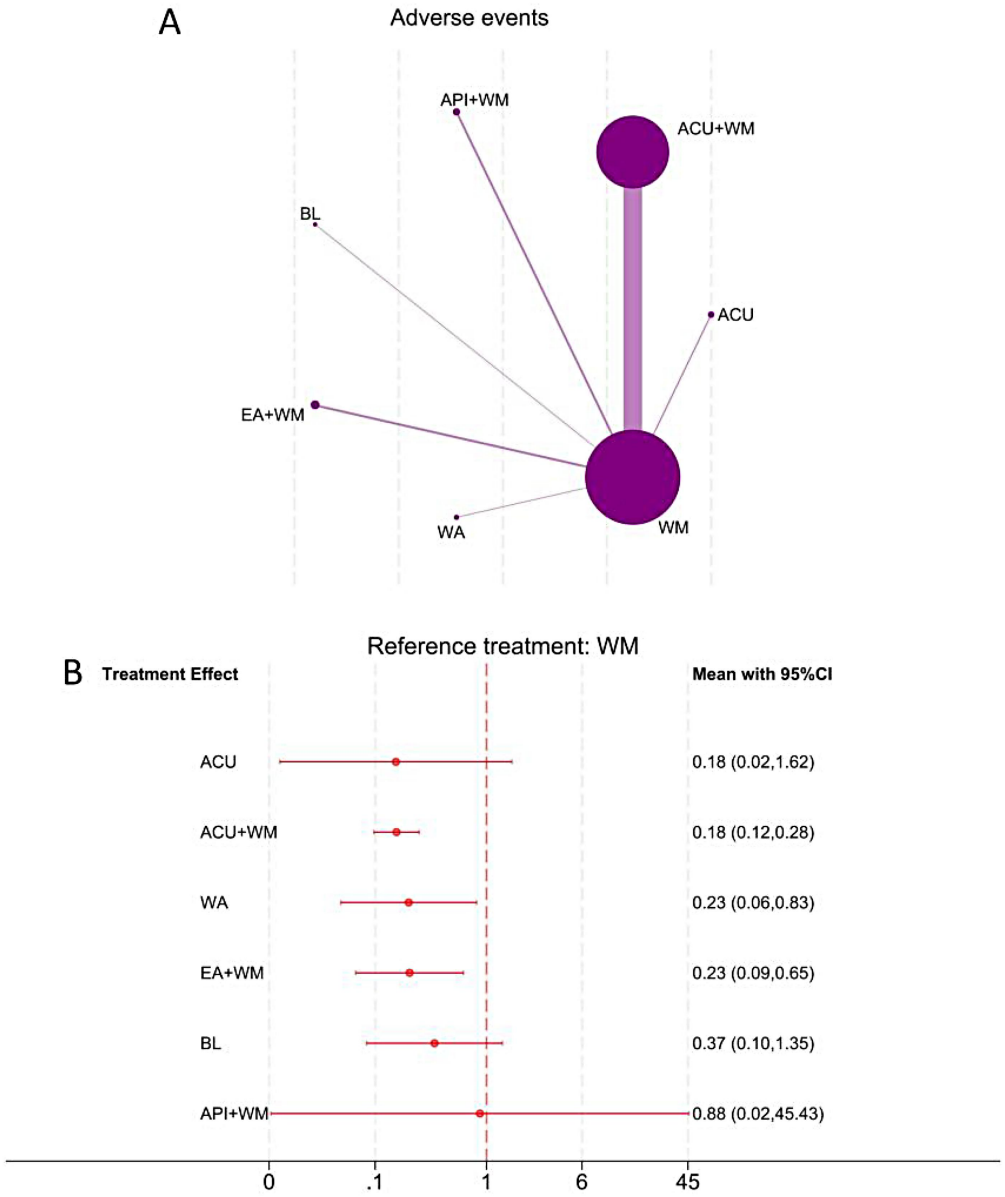
Network map of the effect on AEs, and forest plot of network effect sizes compared with WM. **(A)** Network map of the effect on AEs. **(B)** Forest plot of network effect sizes compared with WM.

For AEs, this network meta-analysis included 24 RCTs, covering seven interventions and a total of 1,653 participants. The evidence network (Figure 6A) formed a star-shaped structure, with conventional Western medicine as the central comparator directly linked to all six acupuncture-based interventions. As shown in Figure 6B, three treatments demonstrated statistically significant benefits. Acupuncture combined with conventional Western medicine demonstrated the greatest benefit (OR=0.18, 95% CI: 0.12–0.28; SUCRA = 73.8%; CINeMA: moderate certainty), followed by moxibustion (OR=0.23, 95% CI: 0.06–0.83; SUCRA = 62.3%; CINeMA: moderate certainty) and electroacupuncture combined with conventional Western medicine (OR=0.23, 95% CI: 0.09–0.65; SUCRA = 61.1%; CINeMA: moderate certainty). No statistically significant differences were observed among the active interventions in the league table. Detailed comparisons of AEs are presented in the SUCRA rankings (Supplementary Appendix S7, Figure S7.5, Table S7.5) and the league table (Supplementary Appendix S8, Table S8.5). AEs associated with acupuncture-related therapies were generally mild and transient, most commonly reflecting local stimulation (e.g., dizziness, fatigue, and localized skin reactions), with few systemic effects reported. In contrast, conventional Western medicine was more frequently associated with central nervous system and gastrointestinal AEs, such as drowsiness, dizziness, nausea, and vomiting, and some studies reported more serious outcomes, including abnormal liver function. These findings indicate that acupuncture-based therapies may have a more favorable safety profile, particularly in reducing drug-related adverse effects.

### 3.5 Sensitivity analyses

Sensitivity analyses confirmed the stability of the results. No single study had a disproportionate influence on the overall estimates, and the findings remained consistent with the primary analysis (Supplementary Appendix 11).

### 3.6 Regression analysis

The results showed no significant association between treatment duration and the estimated effects, and the overall conclusions were consistent with those of the primary analysis (Supplementary Appendix 12). However, given the moderate-to-high heterogeneity observed in some outcomes and the generally limited certainty of the evidence, these findings should be interpreted with caution.

## 4 Discussion

This NMA systematically evaluated the efficacy and safety of different acupuncture modalities and their combination therapies for PTN. The results showed that bloodletting therapy was identified as a potentially effective intervention for improving TER and reducing VAS scores. In contrast, acupuncture combined with conventional Western medicine performed better in reducing AF, improving TCMSS, and decreasing the risk of AEs. This suggests that integrative therapy may provide better control of disease recurrence while also reducing treatment-related side effects. The evidence network displayed a typical star-shaped structure, with conventional Western medicine as the central comparator. Although most acupuncture-based interventions were more effective than Western medicine alone, direct comparisons between different acupuncture modalities were rarely statistically significant. Moreover, the certainty of evidence for several key outcomes, as assessed using the CINeMA framework, was low or very low, highlighting the need for more robust studies. Moreover, although acupuncture was associated with a statistically significant improvement in TCMSS, the magnitude of this effect was small (SMD = 0.15). Based on commonly accepted thresholds for interpreting standardized mean differences, such an effect is considered trivial to small. Therefore, the clinical significance of this finding should be interpreted cautiously, as statistical significance does not necessarily imply a meaningful benefit for patients.

The modest effect size may be attributable to several factors, including variability in syndrome classification across studies, differences in outcome assessment methods, and the subjective nature of TCMSS evaluation. These sources of heterogeneity may have influenced the observed treatment effects. Further well-designed, high-quality clinical trials are needed to determine whether the statistically significant improvements observed in TCMSS translate into meaningful clinical benefits for patients.

Previous meta-analyses have reported beneficial effects of acupuncture in patients with PTN. Studies by Wei and Ang (76, 77) show that, compared with conventional Western medicine such as CBZ, acupuncture significantly reduces pain intensity, as measured by the VAS, and is associated with fewer adverse events. Some evidence further suggests that acupuncture may offer advantages over both pharmacotherapy and microvascular decompression in terms of long-term cost-effectiveness and patient tolerability (78). Despite these findings, earlier studies have generally treated acupuncture as a single, uniform intervention or have focused on comparisons with specific techniques, such as electroacupuncture. As a result, they have not provided a comprehensive evaluation of the relative effects of different acupuncture modalities and combination strategies (79). The present study addresses this gap by offering a more detailed comparison across multiple acupuncture-based interventions. Our results indicate that bloodletting therapy shows a stronger effect in reducing VAS scores. This finding may be related to the proposed mechanism whereby bloodletting therapy improves local microcirculation. In addition, existing evidence highlights limitations of CBZ and similar drugs, including drug resistance and high recurrence rates (80). In contrast, our findings suggest that acupuncture combined with conventional Western medicine appeared to be the most effective intervention for reducing attack frequency, indicating that integrative approaches may better interrupt the cycle of pain and spasm.

Differences in efficacy across acupuncture modalities are likely driven by their distinct mechanisms of action. PTN is commonly linked to neurovascular compression, which can result in local ischemia and inflammation. Bloodletting therapy may provide relief by reducing tissue pressure through mechanical decompression, thereby improving microcirculation around affected nerve endings and alleviating both compression and ischemic injury (81, 82). Neurogenic inflammation also plays a key role in pain sensitization in PTN. Bloodletting therapy may help reduce the local accumulation of accumulated pain-related mediators—such as substance P, calcitonin gene-related peptide, prostaglandin E2, and bradykinin—thereby interrupting their ongoing activation of peripheral nociceptors and contributing to pain reduction (83, 84). In addition, as an intense noxious stimulus, bloodletting therapy may activate endogenous inhibitory pathways. By engaging brainstem structures, it can trigger diffuse noxious inhibitory control, a process in which one painful stimulus suppresses another. This mechanism allows for widespread inhibition of pain signaling in both the spinal cord and the trigeminal system (85–87). Furthermore, strong needling and bloodletting stimulation may activate central pain-modulating systems, including the hypothalamic–pituitar*y* axis and the periaqueductal gray, thereby promoting the release of endogenous opioids. These substances—including *β*-endorphin, enkephalins, and dynorphins—interact with opioid receptors to produce an effective analgesic response (10, 88).

The benefits of combining acupuncture combined with conventional Western medicine likely reflect complementary mechanisms of action. Drugs such as carbamazepine reduce pain by blocking voltage-gated sodium channels (NaV1.3 and NaV1.7), thereby suppressing abnormal high-frequency discharges in the trigeminal ganglion (49, 89). In contrast, acupuncture primarily influences central pain regulation by enhancing descending inhibitory pathways—through increased serotonin and norepinephrine activity—and by improving the emotional and cognitive aspects of pain, including reductions in anxiety and depression (90–92). Together, these peripheral and central effects may reduce overall neuronal excitability and lower the likelihood of recurrent abnormal discharges (93). In addition, acupuncture's broader regulatory effects may help relieve symptoms associated with chronic pain, such as poor sleep and emotional distress, contributing to improvements in TCMSS (94, 95). Acupuncture may also allow for lower doses of medication in clinical practice. This dose-sparing effect can reduce the risk of AEs, such as dizziness, drowsiness, and liver dysfunction, thereby improving the safety of long-term treatment (96).

A key strength of this study is the integration of both direct and indirect evidence across a range of acupuncture interventions. By using a network meta-analysis, we were able to compare treatments and rank their effectiveness, which may help inform clinical decision-making. However, several limitations should be noted. The evidence network was mainly star-shaped, with limited direct comparisons between treatments, which may reduce the reliability of indirect estimates. The quality of the included randomized controlled trials varied. Because blinding is difficult in acupuncture research, some degree of bias cannot be ruled out (97). In addition, many studies did not clearly report how treatment allocation was concealed and were not prospectively registered. These issues increase the risk of bias and weaken confidence in the findings, as well as the overall robustness of the conclusions. Beyond study design, differences in treatment approaches may also have affected the results. Variations in acupoint selection, needling methods, treatment duration, and bloodletting sites could have contributed to inconsistent outcomes. Of note, acupoint injection combines physical stimulation of acupuncture points with the pharmacological effects of injected agents. Although it is often classified as an acupuncture-related therapy in clinical practice, its drug component may confound the assessment of treatment effects. Some outcome measures, such as the TER, are subjective, and most studies did not include long-term follow-up. Finally, all participants included in this study were adults with primary trigeminal neuralgia; therefore, the findings are limited to adult populations. As adolescents and children may differ from adults in physiological development, disease characteristics, and responses to acupuncture and pharmacological treatments, treatment approaches and dosing regimens established for adults should not be directly applied to these younger populations.

Future research should prioritize large, multicenter, high-quality randomized controlled trials and improve the standardization of acupuncture treatment protocols to enhance the reliability and comparability of results. In parallel, the use of objective assessment tools, such as functional magnetic resonance imaging (fMRI) and validated biomarkers, is encouraged to provide a more comprehensive evaluation of treatment effects and to better clarify underlying mechanisms. Moreover, direct comparisons between different acupuncture approaches are needed, along with well-designed studies involving different age groups, to better define their effectiveness and applicability. Future studies should also examine dose–response relationships, refine combination treatment strategies, and distinguish the respective effects of acupoint stimulation and drug therapy. Together, these efforts will strengthen the evidence base for more precise treatment of primary trigeminal neuralgia.

## 5 Conclusion

In summary, bloodletting therapy showed potential benefits in improving the TER and reducing pain intensity measured by the VAS. Acupuncture combined with conventional Western medicine may also provide benefits in reducing AF, improving TCMSS, and maintaining a favorable safety profile. However, these findings should be interpreted cautiously because several outcomes showed moderate-to-high heterogeneity, and the certainty of evidence assessed by CINeMA ranged from very low to moderate. Therefore, further large-scale, multicenter, high-quality randomized controlled trials are needed to validate the efficacy and safety of different acupuncture-related therapies for primary trigeminal neuralgia.

## Data Availability

The datasets presented in this study can be found in online repositories. The names of the repository/repositories and accession number(s) can be found in the article/Supplementary Material.
