# Supplementary material for: Efficacy and safety of different acupuncture-related therapies for primary trigeminal neuralgia: a systematic review and network meta-analysis
Source: Front Pain Res (Lausanne). 2026 Jul 14;7:1854738. doi: 10.3389/fpain.2026.1854738 (PMC13410674; doi:10.3389/fpain.2026.1854738)
Supplement: Supplementary file 1 [file Datasheet1.docx]

**Supplementary Appendix**

Efficacy and Safety of Different Acupuncture-Related Therapies for Primary Trigeminal Neuralgia: A Systematic Review and Network Meta-analysis

[*Appendix 1: PRISMA-NMA checklist*](#bookmark1) *2*

[*Appendix 2: Search strategy*](#bookmark2) *6*

[*Appendix 3:Characteristics of included studies*](#bookmark3)  *9*

[*Appendix 4: List of data extracted from the included randomized clinical trials*](#bookmark4) *19*

[*Appendix 5: Risk of bias of randomized clinical trials*](#bookmark5) *20*

[*Appendix 6: Evaluation of inconsistency and heterogeneity*](#bookmark7) *24*

*Appendix 7:SUCRA and cumulative probability plots 27*

[*Appendix 8: Pooled efficacy estimates of different acupuncture therapies for primary trigeminal neuralgia from a network meta-analysis of 58 trials*](#bookmark10) *31*

[*Appendix 9: CINeMA Assessment*](#bookmark11) *36*

[*Appendix 10: Funnel plotsSensitivity analyses*](#bookmark12) *48*

*Appendix 11: Sensitivity analyses 52*

*Appendix 12: Regression analyses 54*

**Appendix 1: PRISMA-NMA checklist**

| **Section/Topic** | **Item #** | **Checklist Item** | **Reported on Page #** |
| --- | --- | --- | --- |
| **TITLE** |  |  |  |
| Title | 1 | Identify the report as a systematic review *incorporating a network meta-analysis (or related form of meta-analysis).* | 1 |
|  |  |  |  |
| **ABSTRACT** |  |  |  |
| Structured summary | 2 | Provide a structured summary including, as applicable:  **Background:** main objectives  **Methods:** data sources; study eligibility criteria, participants, and interventions; study appraisal; and *synthesis methods, such as network meta-analysis.*  **Results:** number of studies and participants identified; summary estimates with corresponding confidence/credible intervals; *treatment rankings may also be discussed. Authors may choose to summarize pairwise comparisons against a chosen treatment included in their analyses for brevity.*  **Discussion/Conclusions:** limitations; conclusions and implications of findings.  **Other:** primary source of funding; systematic review registration number with registry name. | 1 |
|  |  |  |  |
| **INTRODUCTION** |  |  |  |
| Rationale | 3 | Describe the rationale for the review in the context of what is already known*, including mention of why a network meta-analysis has been conducted.* | ***2*** |
| Objectives | 4 | Provide an explicit statement of questions being addressed, with reference to participants, interventions, comparisons, outcomes, and study design (PICOS). | 3 |
|  |  |  |  |
| **METHODS** |  |  |  |
| Protocol and registration | 5 | Indicate whether a review protocol exists and if and where it can be accessed (e.g., Web address); and, if available, provide registration information, including registration number. | 3 |
| Eligibility criteria | 6 | Specify study characteristics (e.g., PICOS, length of follow-up) and report characteristics (e.g., years considered, language, publication status) used as criteria for eligibility, giving rationale. *Clearly describe eligible treatments included in the treatment network, and note whether any have been clustered or merged into the same node (with justification).* | ***4*** |
| Information sources | 7 | Describe all information sources (e.g., databases with dates of coverage, contact with study authors to identify additional studies) in the search and date last searched. | 3 |
| Search | 8 | Present full electronic search strategy for at least one database, including any limits used, such that it could be repeated. | **Appendix 2** |
| Study selection | 9 | State the process for selecting studies (i.e., screening, eligibility, included in systematic review, and, if applicable, included in the meta-analysis). | 4 |
| Data collection process | 10 | Describe method of data extraction from reports (e.g., piloted forms, independently, in duplicate) and any processes for obtaining and confirming data from investigators. | 4 |
| Data items | 11 | List and define all variables for which data were sought (e.g., PICOS, funding sources) and any assumptions and simplifications made. | 4 |
| **Geometry of the network** | **S1** | Describe methods used to explore the geometry of the treatment network under study and potential biases related to it. This should include how the evidence base has been graphically summarized for presentation, and what characteristics were compiled and used to describe the evidence base to readers. | ***4*** |
| Risk of bias within individual studies | 12 | Describe methods used for assessing risk of bias of individual studies (including specification of whether this was done at the study or outcome level), and how this information is to be used in any data synthesis. | 5 |
| Summary measures | 13 | State the principal summary measures (e.g., risk ratio, difference in means). *Also describe the use of additional summary measures assessed, such as treatment rankings and surface under the cumulative ranking curve (SUCRA) values, as well as modified approaches used to present summary findings from meta-analyses.* | 5 |
| Planned methods of analysis | 14 | Describe the methods of handling data and combining results of studies for each network meta-analysis. This should include, but not be limited to:   - *Handling of multi-arm trials;* - *Selection of variance structure;* - *Selection of prior distributions in Bayesian analyses; and* - *Assessment of model fit.* | 6 |
| **Assessment of Inconsistency** | **S2** | Describe the statistical methods used to evaluate the agreement of direct and indirect evidence in the treatment network(s) studied. Describe efforts taken to address its presence when found. | 7 |
| Risk of bias across studies | 15 | Specify any assessment of risk of bias that may affect the cumulative evidence (e.g., publication bias, selective reporting within studies). | **7** |
| Additional analyses | 16 | Describe methods of additional analyses if done, indicating which were pre-specified. This may include, but not be limited to, the following:   - Sensitivity or subgroup analyses; - Meta-regression analyses; - *Alternative formulations of the treatment network; and* - *Use of alternative prior distributions for Bayesian analyses (if applicable).* | ***7*** |
| **RESULTS†** |  |  |  |
| Study selection | 17 | Give numbers of studies screened, assessed for eligibility, and included in the review, with reasons for exclusions at each stage, ideally with a flow diagram. | 6 |
| **Presentation of network structure** | **S3** | Provide a network graph of the included studies to enable visualization of the geometry of the treatment network. | ***8*** |
| **Summary of network geometry** | **S4** | Provide a brief overview of characteristics of the treatment network. This may include commentary on the abundance of trials and randomized patients for the different interventions and pairwise comparisons in the network, gaps of evidence in the treatment network, and potential biases reflected by the network structure. | ***7*** |
| Study characteristics | 18 | For each study, present characteristics for which data were extracted (e.g., study size, PICOS, follow-up period) and provide the citations. | 7 |
| Risk of bias within studies | 19 | Present data on risk of bias of each study and, if available, any outcome level assessment. | 7 |
| Results of individual studies | 20 | For all outcomes considered (benefits or harms), present, for each study: 1) simple summary data for each intervention group, and 2) effect estimates and confidence intervals. *Modified approaches may be needed to deal with information from larger networks.* | ***Appendix3*** |
| Synthesis of results | 21 | Present results of each meta-analysis done, including confidence/credible intervals. *In larger networks, authors may focus on comparisons versus a particular comparator (e.g. placebo or standard care), with full findings presented in an appendix. League tables and forest plots may be considered to summarize pairwise comparisons.* If additional summary measures were explored (such as treatment rankings), these should also be presented. | 8 |
| **Exploration for inconsistency** | **S5** | Describe results from investigations of inconsistency. This may include such information as measures of model fit to compare consistency and inconsistency models, *P* values from statistical tests, or summary of inconsistency estimates from different parts of the treatment network. | ***Appendix6*** |
| Risk of bias across studies | 22 | Present results of any assessment of risk of bias across studies for the evidence base being studied. | ***Appendix10*** |
| Results of additional analyses | 23 | Give results of additional analyses, if done (e.g., sensitivity or subgroup analyses, meta-regression analyses*, alternative network geometries studied, alternative choice of prior distributions for Bayesian analyses,* and so forth). | 15 |
| **DISCUSSION** |  |  |  |
| Summary of evidence | 24 | Summarize the main findings, including the strength of evidence for each main outcome; consider their relevance to key groups (e.g., healthcare providers, users, and policy-makers). | 15 |
| Limitations | 25 | Discuss limitations at study and outcome level (e.g., risk of bias), and at review level (e.g., incomplete retrieval of identified research, reporting bias). *Comment on the validity of the assumptions, such as transitivity and consistency. Comment on any concerns regarding network geometry (e.g., avoidance of certain comparisons).* | 18 |
| Conclusions | 26 | Provide a general interpretation of the results in the context of other evidence, and implications for future research. | 18 |
| **FUNDING** |  |  |  |
| Funding | 27 | Describe sources of funding for the systematic review and other support (e.g., supply of data); role of funders for the systematic review. This should also include information regarding whether funding has been received from manufacturers of treatments in the network and/or whether some of the authors are content experts with professional conflicts of interest that could affect use of treatments in the network. | ***18*** |

**Appendix 2: Search strategy**

**Table S1.** Search strategy of PubMed

| # **Searches** |
| --- |
| 1 (Pharmacopuncture[Title/Abstract]) OR ("Acupuncture"[Mesh]) |
| 2 (Moxabustion[Title/Abstract]) OR ("Moxibustion"[Mesh]) |
| 3 "Electroacupuncture"[Mesh] |
| 4 "Needles"[Mesh] |
| 5 (((((Acupuncture Point[Title/Abstract]) OR (Point, Acupuncture[Title/Abstract])) OR (Points, Acupuncture[Title/Abstract])) OR (Acupoints[Title/Abstract])) OR (Acupoint[Title/Abstract])) OR ("Acupuncture Points"[Mesh]))))) |
| 6 #1OR#2OR#3OR#4OR#5 |
| 7 ((((((((((Neuralgia, Trigeminal[Title/Abstract]) OR (Trigeminal Neuralgias[Title/Abstract])) OR (Tic Douloureux[Title/Abstract])) OR (Fothergill Disease[Title/Abstract])) OR (Disease, Fothergill[Title/Abstract])) OR (Trifacial Neuralgia[Title/Abstract])) OR (Neuralgia, Trifacial[Title/Abstract])) OR (Trifacial Neuralgias[Title/Abstract])) OR (Tic Doloureux[Title/Abstract])) OR (Primary trigeminal neuralgia[Title/Abstract])) OR ("Trigeminal Neuralgia"[Mesh]))))))) |
| 8 #6AND#7 |
| 9 (((Clinical Trials, Randomized[Title/Abstract]) OR (Trials, Randomized Clinical[Title/Abstract])) OR (Controlled Clinical Trials, Randomized[Title/Abstract])) OR ("Randomized Controlled Trials as Topic"[Mesh]))) |
| 10 #8AND#9 |

**Table S2.** Search strategy of Web of Science: Science Citation Index Expanded

| # **Searches** |
| --- |
| 1 TS=(Pharmacopuncture) OR TS=(Acupuncture) OR TS=(Moxabustion) OR TS=(Moxibustion) OR TS=(Electroacupuncture) OR TS=(Needles) OR TS=(Acupuncture Point) OR TS=(Point, Acupuncture) OR TS=(Points, Acupuncture) OR TS=(Acupoints) OR TS=(Acupoint) OR TS=(Acupuncture Points) |
| 2 TS=(Neuralgia, Trigeminal)) OR TS=(Trigeminal Neuralgias) OR TS=(Tic Douloureux) OR TS=(Fothergill Disease) OR TS=(Disease, Fothergill) OR TS=(Trifacial Neuralgia) OR TS=(Neuralgia, Trifacial) OR TS=(Trifacial Neuralgias) OR TS=(Tic Doloureux) OR TS=(Primary trigeminal neuralgia) OR TS=(Trigeminal Neuralgia) |
| 3 #1AND#2 |
| 4 TS=(Clinical Trials, Randomized)) OR TS=(Trials, Randomized Clinica) OR TS=(Controlled Clinical Trials, Randomized) OR TS=(Controlled Clinical Trials, Randomized) OR TS=(Randomized Controlled Trials as Topic) |
| 5 #3AND#4 |

**Table S3.** Search strategy of Embase

| # **Searches** |
| --- |
| 1 "Pharmacopuncture":ti,ab,kw OR "Acupuncture:ti,ab,kw OR "Moxabustion":ti,ab,kw OR "Electroacupuncture":ti,ab,kw OR "Needles":ti,ab,kw"Acupuncture Point":ti,ab,kw OR "Point, Acupuncture:ti,ab,kw OR "Points, Acupuncture":ti,ab,kw OR "Acupoints":ti,ab,kw OR "Acupoint":ti,ab,kw OR "Acupuncture Points":ti,ab,kw |
| 2 "(Neuralgia, Trigeminal":ti,ab,kw OR "Trigeminal Neuralgias:ti,ab,kw OR "Tic Douloureux":ti,ab,kw OR "Fothergill Disease":ti,ab,kw OR "Disease, Fothergill":ti,ab,kw"Trifacial Neuralgia":ti,ab,kw OR "Neuralgia, Trifacial:ti,ab,kw OR "Trifacial Neuralgias":ti,ab,kw OR "Tic Doloureux":ti,ab,kw OR "Primary trigeminal neuralgia":ti,ab,kw OR "Trigeminal Neuralgia":ti,ab,kw |
| 3 #1AND#2 |
| 4 "Trials, Randomized Clinica":ti,ab,kw OR "Controlled Clinical Trials, Randomized":ti,ab,kw OR "Controlled Clinical Trials, Randomized":ti,ab,kw"Randomized Controlled Trials as Topic":ti,ab,kw |
| 5 #3AND#4 |

**Table S4.** Search strategy of CochraneLibrary

| #  **Searches** |
| --- |
| 1 "Pharmacopuncture":ti,ab,kw OR "Acupuncture:ti,ab,kw OR "Moxabustion":ti,ab,kw OR "Electroacupuncture":ti,ab,kw OR "Needles":ti,ab,kw"Acupuncture Point":ti,ab,kw OR "Point, Acupuncture:ti,ab,kw OR "Points, Acupuncture":ti,ab,kw OR "Acupoints":ti,ab,kw OR "Acupoint":ti,ab,kw OR "Acupuncture Points":ti,ab,kw |
| 2 "(Neuralgia, Trigeminal":ti,ab,kw OR "Trigeminal Neuralgias:ti,ab,kw OR "Tic Douloureux":ti,ab,kw OR "Fothergill Disease":ti,ab,kw OR "Disease, Fothergill":ti,ab,kw"Trifacial Neuralgia":ti,ab,kw OR "Neuralgia, Trifacial:ti,ab,kw OR "Trifacial Neuralgias":ti,ab,kw OR "Tic Doloureux":ti,ab,kw OR "Primary trigeminal neuralgia":ti,ab,kw OR "Trigeminal Neuralgia":ti,ab,kw |
| 3 #1AND#2 |
| 4 "Trials, Randomized Clinica":ti,ab,kw OR "Controlled Clinical Trials, Randomized":ti,ab,kw OR "Controlled Clinical Trials, Randomized":ti,ab,kw"Randomized Controlled Trials as Topic":ti,ab,kw |
| 5 #3AND#4 |

**Table S5.** Search strategy of CNKI

| **Searches** |
| --- |
| （篇关摘：针刺（精确））OR（篇关摘：针灸（精确））OR（篇关摘：灸（精确））OR（篇关摘：针（精确））OR（篇关摘：电针（精确））OR（篇关摘：穴（精确））AND（篇关摘：原发性三叉神经痛（精确））AND （篇关摘：随机（精确））OR（篇关摘：随机对照试验（精确））OR（篇关摘：RCT（精确）） |

**Table S6.** Search strategy of Wanfang

| **Searches** |
| --- |
| 题名或关键词:(针刺) or 题名或关键词:(针灸) or 题名或关键词:(针刺) or 题名或关键词:(灸) or 题名或关键词:(针) or 题名或关键词:(电针) or 题名或关键词:(穴) and 题名或关键词:(原发性三叉神经痛) and 题名或关键词:(随机) or 题名或关键词:(随机对照试验) or 题名或关键词:(RCT) |

**Table S7.** Search strategy of Weipu

| **Searches** |
| --- |
| [(((((((题名或关键词=针灸 OR 题名或关键词=针刺) OR 题名或关键词=针) OR 题名或关键词=灸) OR 题名或关键词=电针) OR 题名或关键词=穴) AND 题名或关键词=原发性三叉神经痛) AND ((题名或关键词=随机对照试验 OR 题名或关键词=随机对照) OR 题名或关键词=RCT))](http://qikan.cqvip.com/Qikan/search/index?LngMySearHistoryIdGuid=53b4087a-f440-4453-aae6-a18d2c5fdf6a&from=Qikan_Article_History)))))) |

**Appendix 3: Characteristics of included studies**

**Table S3.1: Baseline of characteristics of included studies**

| **Author and Year** | **Basic characteristics of literature** | | | | | | | | **Treatment course** | **Outcome Indicators** |
| --- | --- | --- | --- | --- | --- | --- | --- | --- | --- | --- |
|  | **Number of cases - T** | **Example number - C** | **Age (T)**  **(mean ± SD)，years** | **Age (C)**  **(mean± SD)，years** | **Disease course**  **(T)** | **Disease course**  **(C)** | **I-treatment group** | **I-Control Group** |  |  |
| Li Ziyi 2022 | 30 | 15 | 62.00 ± 3.00 | 64.00 ± 4.00 | 1.75~12.25 | 1.00~6.00 | Trigger point bloodletting therapy | Oral carbamazepine | 8 days | 12 |
| Shangguan Shuhui 2016 | 40 | 40 | 40.75±6.12 | 40.51±6.27 | 1.61±0.77 | 1.54±0.73 | Acupuncture (ACU) | Oral carbamazepine | 30 days | 1 |
| Tian Yangyang 2016 | 30 | 30 | 50.97±9.00 | 51.27±8.5 | 16.43±7.56 | 15.70±7.26 | Electroacupuncture + Carbamazepine | Oral carbamazepine | 21 days | 1234 |
| Hu Yehua 2021 | 30 | 30 | 52.91±4.01 | 52.78±4.10 | 1.63±0.87 | 1.71±0.77 | Electroacupuncture | Oral carbamazepine | 20 days | 12 |
| Ye Sheng 2024 | 40 | 36 | 55.7±6.4 | 56.2±6.1 | 7.24±2.20 | 7.30±2.13 | Acupuncture + Carbamazepine | Pregabalin combined with carbamazepine | 14 days | 12345 |
| Wang Xiaofeng 2018 | 42 | 42 | 58.2±6.1 | 57.3±6.9 | / | / | acupuncture | Oral carbamazepine | 30 days | 14 |
| Wang Yan 2024 | 34 | 34 | 44.30 ± 5.01 | 43.98 ± 4.98 | / | / | Acupuncture + Carbamazepine | Oral carbamazepine | 14 days | 12 |
| Liu Tingting 2020 | 51 | 45 | 49.6±11.4 | 50.3±10.7 | 35.1±7.3 | 34.6±7.8 | Acupoint injection + carbamazepine | Oral carbamazepine | 21 days | 124 |
| Si Shuxi 2018 | 33 | 33 | 57.12±7.89 | 56.36±7.56 | 2.36±0.51 | 2.36±0.51 | Electroacupuncture + Carbamazepine | Oral carbamazepine | 30 days | 124 |
| Feng Shuping 2016 | 109 | 108 | 58.4±4.3 | 58.3±4.2 | 4.9±2.3 | 4.7±2.1 | Acupuncture + Carbamazepine | Oral carbamazepine | 28 days | 1 |
| Luo Hui 2017 | 40 | 43 | 55.00 ± 7.50 | | 3.00 ± 1.00 | | Needle embedding + carbamazepine | Oral carbamazepine | 30 days | 12 |
| Li Binqing 2018 | 33 | 43 | 44.2±14.6 | 43.6±15.8 | 3.0±1.9 | 2.8±1.6 | Trigger point needle + Gabapentin | Gabapentin | 28 days | 14 |
| Chen Guangshun 2019 | 40 | 40 | 44.7±3.5 | 46.2±4.1 | 5.5±1.2 | 6.1±1.6 | Acupuncture + Carbamazepine | Oral carbamazepine | / | 14 |
| Chen Cheng 2025 | 35 | 34 | 47.11±7.08 | 48.06±7.47 | 21.57±5.05 | 22.65±3.99 | Sparrow-pecking moxibustion + Carbamazepine | Oral carbamazepine | 28 days | 125 |
| Yan Fei 2017 | 30 | 30 | 56.07±10.49 | 55.53±10.38 | / | / | Electroacupuncture | Oral carbamazepine | 24 days | 125 |
| Wu Jiali 2019 | 14 | 14 | 45.24±10.78 | 47.06±11.02 | 18.63±9.64 | 17.51±9.15 | warm needle moxibustion | Oral carbamazepine | 30 days | 12 |
| Bai Yang 2021 | 25 | 25 | 49.5±4.4 | 48.4±4.0 | / | / | warm needle moxibustion | Oral carbamazepine | / | 1 |
| Zheng Jiafeng 2017 | 30 | 30 | 50.83±8.23 | 51.23±8.58 | 16.45±7.54 | 15.89±7.83 | Acupoint embedding | Oral carbamazepine | 42 days | 125 |
| Li Yuping 2022 | 44 | 43 | 49.53±5.13 | 50.34±5.22 | 1.21±0.22 | 1.14±0.31 | Acupoint injection (vitamin B12) + carbamazepine | Oral carbamazepine | 40 days | 12 |
| Jiang Ying 2017 | 30 | 30 | 43.03±12.12 | 45.87±11.15 | 42.6±19.71 | 43.17±18.81 | Acupoint injection (lidocaine) + carbamazepine | Oral carbamazepine | 28 days | 125 |
| Niu Qiong 2021 | 36 | 36 | 56.37±3.21 | 56.43±3.25 | 3.45±0.26 | 3.52±0.31 | Acupuncture + Carbamazepine | Oral carbamazepine | 30 days | 123 |
| Bai Chengwu 2020 | 60 | 60 | 59.24±6.5 | 59.15±6.6 | 1.1±0.3 | 1.1±0.4 | Acupuncture + Carbamazepine | Oral carbamazepine | 30 days | 15 |
| Zhu Jianing 2020 | 21 | 21 | 57.16±3.58 | 56.34±3.67 | 9.12±2.55 | 9.34±2.37 | Acupuncture + Carbamazepine | Oral carbamazepine | 30 days | 15 |
| Pan Zhongqiang 2017 | 31 | 30 | 54±11 | 59±11 | 4.74±1.05 | 6.62±1.89 | Acupuncture + Carbamazepine | Oral carbamazepine | 28 days | 123 |
| Wei Yuting 2016 | 16 | 16 | 53.00 ± 11.50 | 51.50 ± 11.75 | 5.13 ± 2.44 | 4.92 ± 2.29 | Acupuncture + Carbamazepine | Oral carbamazepine | 30 days | 1 |
| Liu Xiufen 2020 | 34 | 34 | 53.6±4.5 | | / | | Acupuncture + Carbamazepine | Oral carbamazepine | 31 days | 12 |
| Shen Qinyan2016 | 40 | 40 | 59.57±6.27 | 59.82±6.82 | 7.35±2.19 | 7.21±2.30 | Acupuncture + Carbamazepine | Oral carbamazepine | 30 days | 1234 |
| Ta Yuqin 2020 | 36 | 36 | 48.639±10.393 | 46.694 ±9.913 | / | / | Acupuncture + Carbamazepine | Oral carbamazepine | 31 days | 125 |
| Qin Limei 2021 | 33 | 33 | 54.5±7.8 | 55.2±6.3 | / | / | Acupuncture + Carbamazepine | Oral carbamazepine | 30 days | 12 |
| Jiang Nan 2020 | 28 | 28 | 50.6±2.1 | 58.7±2.3 | 3.2±1.6 | 2.9±1.3 | Acupuncture + Carbamazepine | Oral carbamazepine | 31 days | 1 |
| Wang Ying 2019 | 15 | 15 | 45.8±7.9 | 44.8±7.7 | 2.5±0.8 | 2.6±0.7 | Acupuncture + Carbamazepine | Oral carbamazepine | 90 days | 1 |
| Chen Hui 2021 | 40 | 40 | 43.75±5.89 | 43.28±6.43 | 3.64±1.49 | 3.21±1.52 | Acupuncture + Carbamazepine | Oral carbamazepine | 28 days | 124 |
| Wang Guorong 2020 | 21 | 21 | 46.98±8.12 | 45.61±7.33 | 2.6±0.9 | 2.5±0.8 | Acupuncture + Carbamazepine | Oral carbamazepine | 90 days | 1 |
| Huang Shu 2018 | 32 | 31 | 44.86±6.39 | 43.64±5.47 | 43.64±5.47 | 3.43±1.36 | Acupuncture + Carbamazepine | Oral carbamazepine | 30 days | 124 |
| Liu Yanrong 2016 | 30 | 30 | 42.86±6.28 | 42.67±5.84 | 3.6±1.3 | 3.8±1.1 | Acupuncture + Carbamazepine | Oral carbamazepine | 31 days | 14 |
| Liu Kun 2015 | 42 | 42 | 54.27±3.15 | 53.71±3.40 | 7.63±2.57 | 7.51±2.48 | Acupuncture + Carbamazepine | Oral carbamazepine | 28 days | 1345 |
| Jin Zhaochun 2015 | 37 | 36 | 51.8±4.2 |  | 72.4±15.6 |  | Acupuncture + Carbamazepine | Oral carbamazepine | 30 days | 12 |
| Zhang Wuan 2018 | 30 | 30 | 39.7±4.8 |  | / |  | Acupuncture + Carbamazepine | Oral carbamazepine | 90 days | 12 |
| Ye Qingqing 2020 | 34 | 34 | 62.34±5.49 | 61.22±5.38 | / | / | Acupuncture + Carbamazepine | Oral carbamazepine | / | 123 |
| Wang Juan 2018 | 43 | 43 | 40.06±6.26 | 40.01±6.21 | 10.56±3.26 | 10.51±3.21 | Acupuncture + Carbamazepine | Oral carbamazepine | 90 days | 1 |
| Bai Zhipeng 2021 | 47 | 47 | 60.2±3.0 | 60.0±3.5 | 8.0±1.3 | 7.5±1.2 | Acupuncture + Carbamazepine | Oral carbamazepine | 30 days | 123 |
| Zhou Liping 2016 | 33 | 32 | 42.2±6.1 | 43.5±5.8 | 3.5±1.3 | 3.6±1.2 | Acupuncture + Carbamazepine | Oral carbamazepine | 30 days | 14 |
| Wang Haiyan 2015 | 35 | 35 | 40±5.1 | | 3.5±1.2 | | Acupuncture + Carbamazepine | Oral carbamazepine | 30 days | 14 |
| Xu Jianwei 2019 | 33 | 33 | 54.5±2.2 | 54.8±1.9 | 8.8±0.2 | 8.8±0.2 | Acupuncture + Carbamazepine | Oral carbamazepine | 20 days | 124 |
| Zhao Yang 2021 | 28 | 28 | 48.21±4.37 | 46.34±4.86 | 2.6±1.1 | 2.3±0.9 | Acupuncture + Carbamazepine | Oral carbamazepine | / | 12 |
| Li Yongmei 2018 | 44 | 44 | 42.03±2.56 | 41.20±2.33 | 2.86±1.04 | 3.12±1.21 | Acupuncture + Carbamazepine | Oral carbamazepine | 30 days | 14 |
| Zhou Chunxia 2018 | 15 | 15 | 45.8±7.9 | 44.8±7.7 | 2.5±0.8 | 2.6±0.7 | Acupuncture + Carbamazepine | Oral carbamazepine | 30 days | 14 |
| Wu Chensu 2017 | 48 | 48 | 40.21±3.08 | 42.59±2.11 | 1.48±2.61 | 1.57±3.24 | Acupuncture + Carbamazepine | Oral carbamazepine | 30 days | 1 |
| Xia Weipeng 2015 | 30 | 30 | 25-51 years old | 24-52 years old | 1 month to 5 years | 1 month to 5 years | Acupuncture + Carbamazepine | Oral carbamazepine | 30 days | 124 |
| Hsieh Hui-ling 2016 | 40 | 40 | 40.1±2.05 |  | 2.1±0.2 |  | Acupuncture + Carbamazepine | Oral carbamazepine | 30 days | 14 |
| Yan Hong 2018 | 36 | 36 | 43.13±6.45 | 44.18±7.34 | 3.14±1.16 | 3.14±1.16 | Acupuncture + Carbamazepine | Oral carbamazepine | 30 days | 14 |
| Mudna 2019 | 50 | 50 | 42.6±5.7 | 43.1±5.4 | / | / | Acupuncture + Carbamazepine | Oral carbamazepine | 30 days | 1 |
| Gong Xiaoqian 2019 | 45 | 45 | 58.0±14.0 | 58.0±14.0 | 24.0±20.0 | 23.0±21.0 | Acupuncture + Carbamazepine | Oral carbamazepine | 28 days | 1 |
| Liu Yumei 2019 | 44 | 44 | 48.72±5.27 | 48.34±5.72 | 3.53±1.09 | 3.53±1.09 | Acupuncture + Carbamazepine | Oral carbamazepine | 30 days | 124 |
| Xiao Feng 2016 | 50 | 50 | 54.4±10.8 | 54.2±11.5 | 4.8±3.9 | 4.5±3.2 | Acupuncture + Carbamazepine | Oral carbamazepine | 90 days | 1245 |
| Shi Xueli 2024 | 30 | 30 | 47.35±4.48 | 47.28±5.18 | 5.11±2.21 | 5.22±2.31 | Zhuang medicine thread moxibustion | Oral carbamazepine | 21 days | 124 |
| Hao Wenbin 2019 | 27 | 27 | 45.6±3.4 | 47.2±3.8 | 5.2±1.6 | 5.4±1.8 | Acupuncture + Carbamazepine | Oral carbamazepine | 30 days | 13 |
| Wei Min 2018 | 31 | 31 | 46.01±7.54 | 45.74±7.54 | 2.41±0.25 | 2.37±0.12 | Acupuncture + Carbamazepine | Oral carbamazepine | 30 days | 125 |

**NOTE：①TER，Total Effective Rate；②VAS，Visual Analogue Scale；③AF，Attack frequency；④AE，Adverse events；⑤TCMSS，Traditional Chinese Medicine Syndrome Scoring**

**Table S3.2**：Frequency of acupoint use across included studies

| **Modality Node** | **Studies**  **(n)** | **Most Frequently Used Acupoints**  **(n/N, %)** | **ST36**  **(Zusanli)** | **SP6**  **(Sanyinjiao)** | **KI3**  **(Taixi)** | **GB34**  **(Yanglingquan)** | **LI4 (Hegu)** | **Needle Retention**  **(min)** | **Session Frequency** | **Treatment Duration** |
| --- | --- | --- | --- | --- | --- | --- | --- | --- | --- | --- |
| ACU | 32 | LI4, ST7, GB20, EX-HN5 (≥70%) | Rare | Rare | Rare | Rare | Very common (~90%) | 20–30 | Once daily | 20–30 days |
| EA | 8 | ST7, LI4, GB20 (≥60%) | Rare | Rare | Rare | Rare | Common (~70%) | 20–30 | Once daily / every other day | 3–4 weeks |
| BL | 3 | Ashi points / trigger points (100%) | Not used | Not used | Not used | Not used | Occasionally | Procedure-based (<10 min) | Every 2–3 days | 2–4 weeks |
| ACU + WM | 24 | LI4, ST7, GB20, EX-HN5 (≥80%) | Rare | Rare | Rare | Rare | Very common (~95%) | 20–30 | Once daily | 3–4 weeks |
| EA + WM | 6 | ST7, LI4 (≥70%) | Rare | Rare | Rare | Rare | Common (~80%) | 20–30 | Once daily | 3 weeks |
| API + WM | 3 | LI4, ST7 (≥60%) | Not used | Not used | Not used | Not used | Common | Not applicable | Once daily | 2–4 weeks |
| CAT | 1 | ST7, LI4 | Not used | Not used | Not used | Not used | Common | Not applicable | Every 2–3 days | 2–4 weeks |
| WA | 4 | ST7, LI4 (≥60%) | Rare | Rare | Rare | Rare | Common | 20–30 | Once daily | 3–4 weeks |

**Notes：**ST，stomach meridian；SP，spleen meridian； KI，kidney meridian；GB，gallbladder meridian； LI，large intestine meridian；“Rare” indicates use in <10% of studies； “common” indicates 50–80%； “very common” indicates >80%.

**Table S3.3:**Frequency of acupoint use across included studies

| **Acupoint**  **(WHO Code)** | **Chinese Name** | **Studies Using**  **(n / 47)** | **Usage Rate**  **(%)** | **Meridian** |
| --- | --- | --- | --- | --- |
| LI4 | Hegu | 43 / 47 | 91.5 | Large Intestine Meridian |
| ST7 | Xiaguan | 40 / 47 | 85.1 | Stomach Meridian |
| GB20 | Fengchi | 35 / 47 | 74.4 | Gallbladder Meridian |
| EX-HN5 | Taiyang | 33 / 47 | 70.2 | Extra point |
| EX-HN3 | Yintang | 32 / 47 | 68.1 | Extra point |
| ST2 | Sibai | 30 / 47 | 63.8 | Stomach Meridian |
| ST6 | Jiache | 28 / 47 | 59.6 | Stomach Meridian |
| ST4 | Dicang | 26 / 47 | 55.3 | Stomach Meridian |
| BL10 | Tianzhu | 24 / 47 | 51.1 | Bladder Meridian |
| TE5 | Waiguan | 21 / 47 | 44.6 | Triple Energizer Meridian |
| GB12 | Wangu | 20 / 47 | 42.6 | Gallbladder Meridian |
| LR3 | Taichong | 18 / 47 | 38.3 | Liver Meridian |
| ST44 | Neiting | 16 / 47 | 34 | Stomach Meridian |
| CV24 | Chengjiang | 15 / 47 | 31.9 | Conception Vessel |
| BL2 | Zanzhu | 14 / 47 | 29.8 | Bladder Meridian |

**Notes：**LI, Large Intestine; ST, Stomach; GB, Gallbladder; BL, Bladder; TE, Triple Energizer; LR, Liver; CV, Conception Vessel; EX-HN, Extra Head and Neck points.

**Table S3.4:** Abbreviation

| **Abbreviation** | **Full English name** |
| --- | --- |
| WM | Conventional Western medicine |
| ACU | Acupuncture |
| EA | Electroacupuncture |
| WA | Moxibustion |
| CAT | Acupoint embedding therapy |
| BL | Bloodletting therapy |
| ACU+WM | Acupuncture combined with conventional Western medicine |
| EA+WM | Electroacupuncture combined with conventional Western medicine |
| API+WM | Acupoint injection combined with conventional Western medicine |

**Appendix 4: List of data extracted from the included randomized clinical trials**

| **Data category** | **List of variables** |
| --- | --- |
| Study | Primary author, year of publication, duration of study, total number of patients in each group |
| Patients | Sample size (treatment and control groups), Age, Sex distribution,Disease duration |
| Interventions | Drug class, dose and duration of the primary  intervention and strategies used for implementing them |
| Efficacy outcome | Mean of change in Total Effective Rate (%) , Visual Analogue Scale (point), Attack frequency (%) , Traditional Chinese Medicine Syndrome Scoring (point), with respective standard deviation from baseline |
| Adverse events | Various adverse events reported in included trials |

**Appendix 5: Risk of bias of randomized clinical trials**

**Figure S5:** Overall risk of bias presented as percentage of each risk of bias item across all included studies. Green = Low risk, Red = High risk, Yellow = Some concerns.

**Table S5:** Study-level risk of bias assessment for randomized clinical trials using the Cochrane Risk of Bias 2.0 tool.

| **Unique ID** | **Randomization process** | **Deviations from intended** | **Mising outcome data** | **Measurement of the outcome** | **Selection of the reported result** | **Over all** |
| --- | --- | --- | --- | --- | --- | --- |
|  |  |  |  |  |  |  |
| Li Ziyi 2022 | Low | High | Low | Low | Low | High |
| Shangguan Shuhui 2016 | Low | Some Concerns | Low | Low | Low | Some Concerns |
| Tian Yangyang 2016 | Low | Some Concerns | Low | Low | Low | Some Concerns |
| Hu Yehua 2021 | Low | Some Concerns | Low | Low | Low | Some Concerns |
| Ye Sheng 2024 | Low | Some Concerns | Low | Low | Low | Some Concerns |
| Wang Xiaofeng 2018 | High | Some Concerns | Low | High | Low | High |
| Wang Yan 2024 | Low | Some Concerns | Low | Low | Low | Some Concerns |
| Liu Tingting 2020 | Low | Some Concerns | Low | Low | Low | Some Concerns |
| Si Shuxi 2018 | Low | Some Concerns | Low | Low | Low | Some Concerns |
| Feng Shuping 2016 | Low | Some Concerns | Low | Low | Low | Some Concerns |
| Luo Hui 2017 | Low | Some Concerns | Low | Low | Low | Some Concerns |
| Li Binqing 2018 | Low | Some Concerns | Low | Low | Low | Some Concerns |
| Chen Guangshun 2019 | Low | Some Concerns | Low | Low | Low | Some Concerns |
| Chen Cheng 2025 | Low | Low | Low | Low | Low | Low |
| Yan Fei 2017 | Low | Some Concerns | Low | Low | Low | Some Concerns |
| Wu Jiali 2019 | Low | Some Concerns | Low | Low | Low | Some Concerns |
| Bai Yang 2021 | Low | Some Concerns | Low | Low | Low | Some Concerns |
| Zheng Jiafeng 2017 | Low | Some Concerns | Low | Low | Low | Some Concerns |
| Li Yuping 2022 | Low | Some Concerns | Low | Low | Low | Some Concerns |
| Jiang Ying 2017 | Low | Some Concerns | Low | Low | Low | Some Concerns |
| Niu Qiong 2021 | Low | Some Concerns | Low | Low | Low | Some Concerns |
| Bai Chengwu 2020 | Low | Some Concerns | Low | Low | Low | Some Concerns |
| Zhu Jianing 2020 | High | Some Concerns | Low | High | Low | High |
| Pan Zhongqiang 2017 | Low | Some Concerns | Low | Low | Low | Some Concerns |
| Wei Yuting 2016 | Low | Some Concerns | Low | Low | Low | Some Concerns |
| Liu Xiufen 2020 | Low | Some Concerns | Low | Low | Low | Some Concerns |
| Shen Qinyan2016 | Low | Some Concerns | Low | Low | Low | Some Concerns |
| Ta Yuqin 2020 | Low | Some Concerns | Low | Low | Low | Some Concerns |
| Qin Limei 2021 | Low | Some Concerns | Low | Low | Low | Some Concerns |
| Jiang Nan 2020 | Low | Some Concerns | Low | Low | Low | Some Concerns |
| Wang Ying 2019 | Low | Some Concerns | Low | Low | Low | Some Concerns |
| Chen Hui 2021 | Low | Some Concerns | Low | Low | Low | Some Concerns |
| Wang Guorong 2020 | Low | Some Concerns | Low | Low | Low | Some Concerns |
| Huang Shu 2018 | High | Some Concerns | Low | High | Low | High |
| Liu Yanrong 2016 | Low | Some Concerns | Low | Low | Low | Some Concerns |
| Liu Kun 2015 | Low | Some Concerns | Low | Low | Low | Some Concerns |
| Jin Zhaochun 2015 | Low | Some Concerns | Low | Low | Low | Some Concerns |
| Zhang Wuan 2018 | Low | Some Concerns | Low | Low | Low | Some Concerns |
| Ye Qingqing 2020 | Low | Some Concerns | Low | Low | Low | Some Concerns |
| Wang Juan 2018 | Low | Some Concerns | Low | Low | Low | Some Concerns |
| Bai Zhipeng 2021 | Low | Some Concerns | Low | Low | Low | Some Concerns |
| Zhou Liping 2016 | Low | Some Concerns | Low | Low | Low | Some Concerns |
| Wang Haiyan 2015 | Low | Some Concerns | Low | Low | Low | Some Concerns |
| Xu Jianwei 2019 | Low | Some Concerns | Low | Low | Low | Some Concerns |
| Zhao Yang 2021 | Low | Some Concerns | Low | Low | Low | Some Concerns |
| Li Yongmei 2018 | Low | Some Concerns | Low | Low | Low | Some Concerns |
| Zhou Chunxia 2018 | Low | Some Concerns | Low | Low | Low | Some Concerns |
| Wu Chensu 2017 | High | Some Concerns | Low | High | Low | High |
| Xia Weipeng 2015 | High | Some Concerns | Low | High | Low | High |
| Hsieh Hui-ling 2016 | Low | Some Concerns | Low | Low | Low | Some Concerns |
| Yan Hong 2018 | Low | Some Concerns | Low | Low | Low | Some Concerns |
| Mudna 2019 | Low | Some Concerns | Low | Low | Low | Some Concerns |
| Gong Xiaoqian 2019 | Low | Some Concerns | Low | Low | Low | Some Concerns |
| Liu Yumei 2019 | Low | Some Concerns | Low | Low | Low | Some Concerns |
| Xiao Feng 2016 | Low | Some Concerns | Low | Low | Low | Some Concerns |
| Shi Xueli 2024 | Low | Some Concerns | Low | Low | Low | Some Concerns |
| Hao Wenbin 2019 | Low | Some Concerns | Low | Low | Low | Some Concerns |
| Wei Min 2018 | Low | Some Concerns | Low | Low | Low | Some Concerns |

**Appendix 6: Evaluation of inconsistency and heterogeneity**

**Table S6.1:** Global consistency

| **Clinical outcome** | **Chi square** | **P value** | **tau²** |
| --- | --- | --- | --- |
| Total Effective Rate | 2.30 | 0.1293 | ＜0.04 |
| Visual Analogue Scale | 2.55 | 0.1101 | 0.81 |
| Attack frequency | 2.56 | 0.1093 | 1.56 |
| Traditional Chinese Medicine Syndrome Scoring | 1.22 | 0.2701 | 1.06 |
| Adverse events | 5.03 | 0.0249 | ＜0.04 |

**Table S6.2:** Side-splitting of Total Effective Rate. Inconsistency test between direct and indirect treatment comparisons in mixed treatment comparison.

| **Comparison(A vs B)** | | **Direct** | | **Indirect** | | **Difference** | | |
| --- | --- | --- | --- | --- | --- | --- | --- | --- |
| **A** | **B** | **Coef.** | **Std. Err.** | **Coef.** | **Std. Err.** | **Coef.** | **Std. Err.** | **P>\|z\|** |
| ACU | WM | -0.2158837 | 0.0925214 | -0.0866049 | 1.674521 | -0.1292788 | 1.677077 | 0.939 |
| ACU+WM | WM | -0.1702861 | 0.0154892 | -0.4361053 | 3.732828 | 0.2658191 | 3.732899 | 0.943 |
| API+WM | WM | -0.1589055 | 0.0482504 | -0.4156469 | 11.91858 | 0.2567414 | 11.91881 | 0.983 |
| BL | WM | -0.6931472 | 0.2816978 | -0.1024595 | 33.80584 | -0.5906877 | 33.8092 | 0.986 |
| CAT | WM | -0.1541507 | 0.1072219 | -0.4250608 | 24.80885 | 0.2709101 | 24.80932 | 0.991 |
| EA+WM | WM | -0.3247861 | 0.0972222 | -0.4235159 | 24.30665 | 0.0987299 | 24.30708 | 0.997 |
| IN+WM | WM | -0.1040694 | 0.1266274 | -0.4320446 | 35.64778 | 0.3279752 | 35.64808 | 0.993 |
| WA | WM | -0.1237542 | 0.0829853 | -0.4895466 | 15.7305 | 0.3657924 | 15.73075 | 0.981 |
| WA+WM | WM | -0.1861238 | 0.1227075 | -0.4196531 | 30.47131 | 0.2335293 | 30.47179 | 0.994 |

**Table S6.3:** Side-splitting of Visual Analogue Scale. Inconsistency test between direct and indirect treatment comparisons in mixed treatment comparison.

| **Comparison(A vs B)** | | **Direct** | | **Indirect** | | **Difference** | | |
| --- | --- | --- | --- | --- | --- | --- | --- | --- |
| **A** | **B** | **Coef.** | **Std. Err.** | **Coef.** | **Std. Err.** | **Coef.** | **Std. Err.** | **P>\|z\|** |
| ACU+WM | WM | 1.586382 | 0.2285893 | 1.277103 | 27.72356 | 0.3092785 | 27.72451 | 0.991 |
| API+WM | WM | 1.620798 | 0.541821 | 1.551922 | 115.4431 | 0.0688759 | 115.4446 | 1 |
| BL | WM | 2.7 | 1.127881 | 0.4727097 | 200.0132 | 2.22729 | 200.0187 | 0.991 |
| CAT | WM | 1.4 | 0.9915401 | 1.772723 | 199.9953 | -0.3727234 | 199.9986 | 0.999 |
| EA | WM | 0.41 | 0.9035219 | 2.762738 | 200.0073 | -2.352738 | 200.0094 | 0.991 |
| EA+WM | WM | 1.091922 | 0.5721265 | 2.080806 | 115.4908 | -0.9888831 | 115.4928 | 0.993 |
| IN+WM | WM | 1.2 | 0.9776688 | 1.972725 | 199.9988 | -0.7727251 | 200.0019 | 0.997 |
| WA | WM | 0.6271189 | 0.6882097 | 2.545622 | 141.4323 | -1.918503 | 141.4344 | 0.989 |
| WA+WM | WM | 1.5 | 0.9389606 | 1.672724 | 200.0123 | -0.1727244 | 200.0149 | 0.999 |

**Table S6.4:** Side-splitting of Attack frequency. Inconsistency test between direct and indirect treatment comparisons in mixed treatment comparison.

| **Comparison(A vs B)** | | **Direct** | | **Indirect** | | **Difference** | | |
| --- | --- | --- | --- | --- | --- | --- | --- | --- |
| **A** | **B** | **Coef.** | **Std. Err.** | **Coef.** | **Std. Err.** | **Coef.** | **Std. Err.** | **P>\|z\|** |
| ACU+WM | WM | 3.023138 | 0.5326191 | -2.339986 | 100.0362 | 5.363125 | 100.0376 | 0.957 |
| EA+WM | WM | -2.34 | 1.460765 | 8.386279 | 200.0102 | -10.72628 | 200.0184 | 0.957 |

**Table S6.5:** Side-splitting of Traditional Chinese Medicine Syndrome Scoring. Inconsistency test between direct and indirect treatment comparisons in mixed treatment comparison.

| **Comparison(A vs B)** | | **Direct** | | **Indirect** | | **Difference** | | |
| --- | --- | --- | --- | --- | --- | --- | --- | --- |
| **A** | **B** | **Coef.** | **Std. Err.** | **Coef.** | **Std. Err.** | **Coef.** | **Std. Err.** | **P>\|z\|** |
| ACU+WM | WM | 1.885419 | 0.4059924 | 0.2805438 | 50.00371 | 1.604875 | 50.00536 | 0.974 |
| API+WM | WM | 0.3513232 | 1.063107 | 3.419354 | 200.0023 | -3.068031 | 200.0055 | 0.988 |
| CAT | WM | -0.8543021 | 1.065685 | 4.625039 | 200.0336 | -5.479341 | 200.0368 | 0.978 |
| EA+WM | WM | 0.4524411 | 1.063453 | 3.318237 | 200.0033 | -2.865796 | 200.0065 | 0.989 |
| WA+WM | WM | 1.172713 | 1.06353 | 2.597937 | 200.0081 | -1.425224 | 200.0113 | 0.994 |

**Table S6.6:** Side-splitting of Adverse events. Inconsistency test between direct and indirect treatment comparisons in mixed treatment comparison.

| **Comparison(A vs B)** | | **Direct** | | **Indirect** | | **Difference** | | |
| --- | --- | --- | --- | --- | --- | --- | --- | --- |
| **A** | **B** | **Coef.** | **Std. Err.** | **Coef.** | **Std. Err.** | **Coef.** | **Std. Err.** | **P>\|z\|** |
| ACU | WM | 1.712092 | 1.118668 | 0.6282069 | 19.51265 | 1.083885 | 19.54469 | 0.956 |
| ACU+WM | WM | 1.697224 | 0.2174551 | 3.852867 | 46.00343 | -2.155643 | 46.00338 | 0.963 |
| API+WM | WM | 0.1238695 | 2.010322 | 3.414142 | 629.3558 | -3.290273 | 629.359 | 0.996 |
| BL | WM | 0.9808293 | 0.6531365 | 3.133484 | 130.4996 | -2.152655 | 130.502 | 0.987 |
| EA+WM | WM | 1.449868 | 0.5178576 | 3.615335 | 117.1206 | -2.165467 | 117.121 | 0.985 |
| WA | WM | 1.466337 | 0.6537205 | 3.671688 | 143.0723 | -2.205351 | 143.0728 | 0.988 |

**Appendix 7: SUCRA and cumulative probability plots**

**Figure S7.1:** Cumulative ranking curve plots of different acupuncture techniques for Total Effective Rate in range network. Higher surface under the curve reflects higher probability of association with Total Effective Rate.


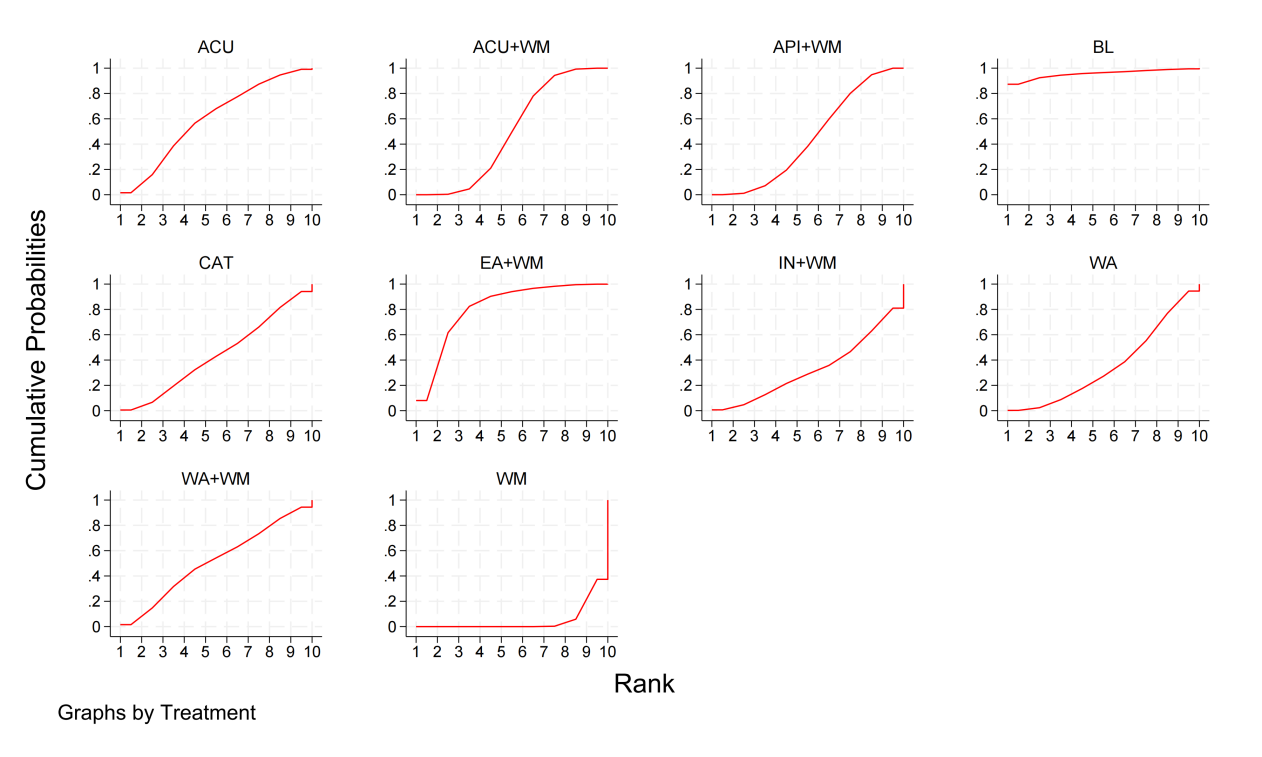


**Table S7.1:** SUCRA of the effects of different acupuncture techniques on Total Effective Rate.

| **Treatment** | **SUCRA** | **PrBest** | **MeanRank** |
| --- | --- | --- | --- |
| ACU | 60.2 | 1.6 | 4.6 |
| ACU+WM | 48.9 | 0 | 5.6 |
| API+WM | 44.7 | 0.1 | 6 |
| BL | 95.6 | 87.3 | 1.4 |
| CAT | 44.2 | 0.5 | 6 |
| EA+WM | 81.3 | 8.0 | 2.7 |
| IN+WM | 32.8 | 0.6 | 7.0 |
| WA | 35.8 | 0.2 | 6.8 |
| WA+WM | 51.7 | 1.6 | 5.3 |
| WM | 4.8 | 0 | 9.6 |

Abbreviations: SUCRA, surface under the cumulative ranking curve.

**Figure S7.2:** Cumulative ranking curve plots of different acupuncture techniques for Visual Analogue Scale in range network. Higher surface under the curve reflects higher probability of association with Visual Analogue Scale.


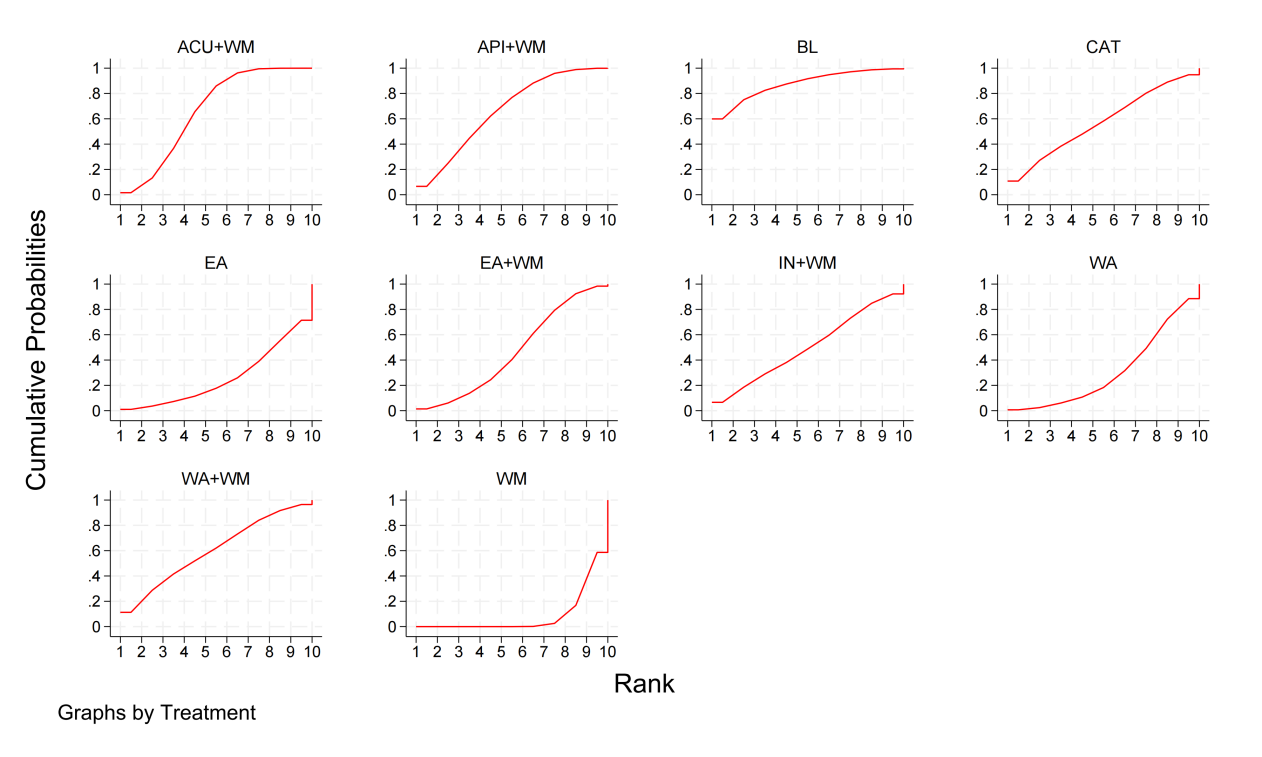


**Table S7.2:** SUCRA of the effects of different acupuncture techniques on Visual Analogue Scale.

| **Treatment** | **SUCRA** | **PrBest** | **MeanRank** |
| --- | --- | --- | --- |
| ACU+WM | 66.5 | 1.6 | 4 |
| API+WM | 66.5 | 6.6 | 4 |
| BL | 87.4 | 59.9 | 2.1 |
| CAT | 57.3 | 10.8 | 4.8 |
| EA | 25.9 | 1 | 7.7 |
| EA+WM | 46.4 | 1.4 | 5.8 |
| IN+WM | 50.1 | 6.6 | 5.5 |
| WA | 31.1 | 0.7 | 7.2 |
| WA+WM | 60.1 | 11.3 | 4.6 |
| WM | 8.7 | 0 | 9.2 |

Abbreviations: SUCRA, surface under the cumulative ranking curve.

**Figure S7.3:** Cumulative ranking curve plots of different acupuncture techniques for Attack frequency in range network. Higher surface under the curve reflects higher probability of association with Attack frequency.


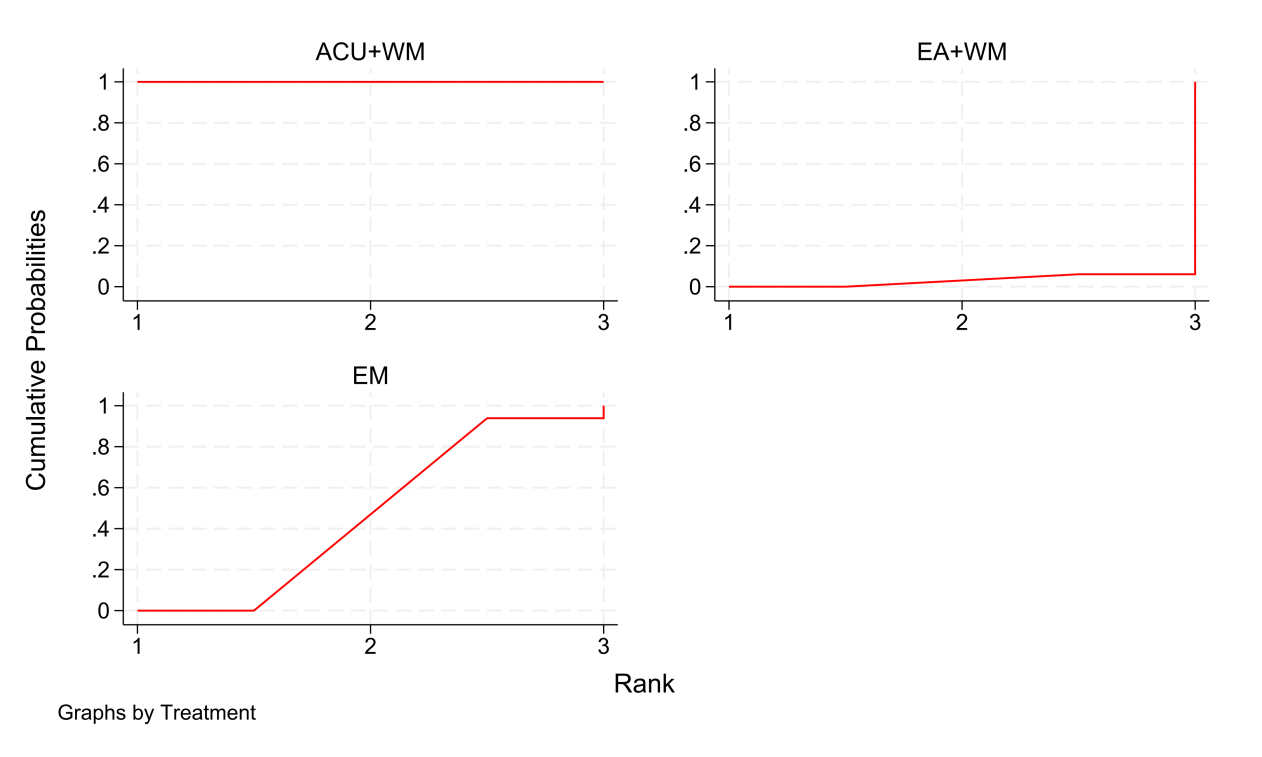


**Table S7.3:** SUCRA of the effects of different acupuncture techniques on Attack frequency.

| **Treatment** | **SUCRA** | **PrBest** | **MeanRank** |
| --- | --- | --- | --- |
| ACU+WM | 100 | 100 | 1 |
| EA+WM | 2.7 | 0 | 2.9 |
| WM | 47.5 | 0 | 2.1 |

Abbreviations: SUCRA, surface under the cumulative ranking curve.

**Figure S7.4:** Cumulative ranking curve plots of different acupuncture techniques for Traditional Chinese Medicine Syndrome Scoring in range network. Higher surface under the curve reflects higher probability of association with Traditional Chinese Medicine Syndrome Scoring.


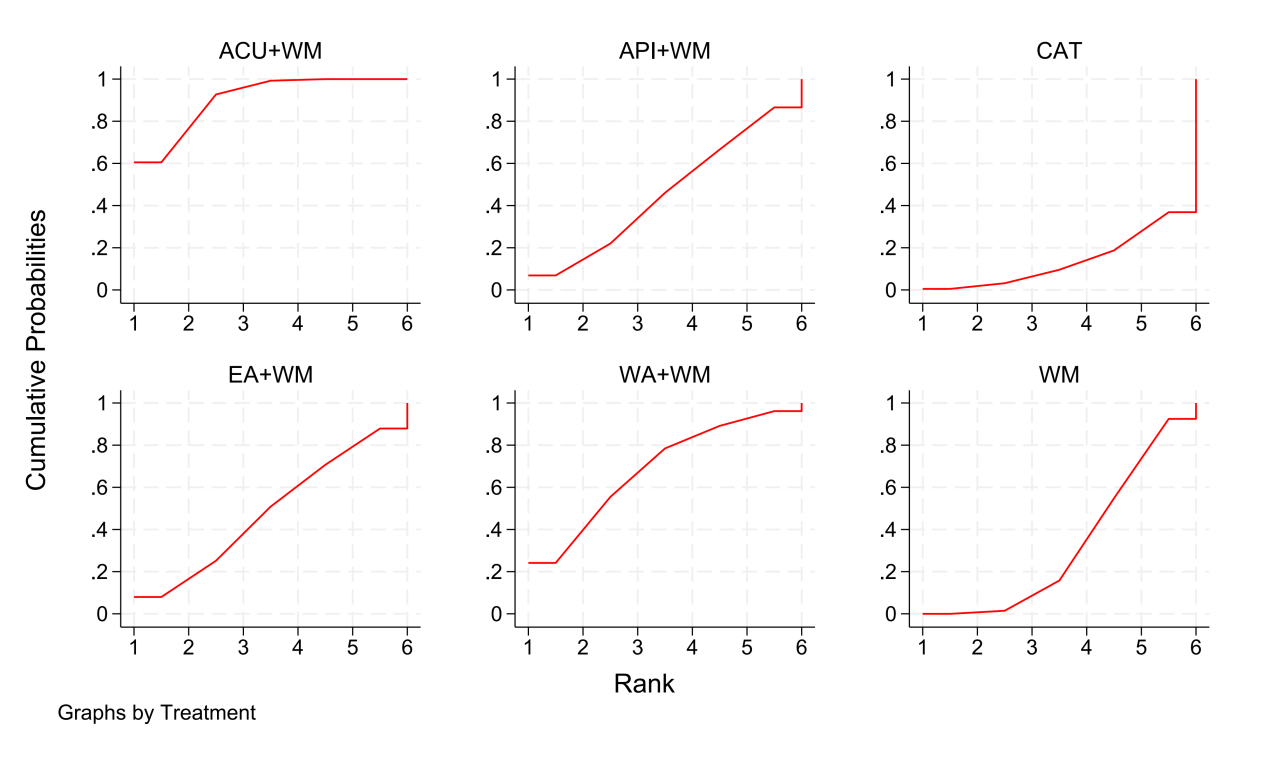


**Table S7.4:** SUCRA of the effects of different acupuncture techniques on Traditional Chinese Medicine Syndrome Scoring.

| **Treatment** | **SUCRA** | **PrBest** | **MeanRank** |
| --- | --- | --- | --- |
| ACU+WM | 90.7 | 61.6 | 1.5 |
| API+WM | 45.6 | 6.5 | 3.7 |
| CAT | 13.9 | 0.4 | 5.3 |
| EA+WM | 49.1 | 7.9 | 3.5 |
| WA+WM | 67.9 | 23.6 | 2.6 |
| WM | 32.8 | 0 | 4.4 |

Abbreviations: SUCRA, surface under the cumulative ranking curve.

**Figure S7.5:** Cumulative ranking curve plots of different acupuncture techniques for Adverse events in range network. Higher surface under the curve reflects higher probability of association with Adverse events.


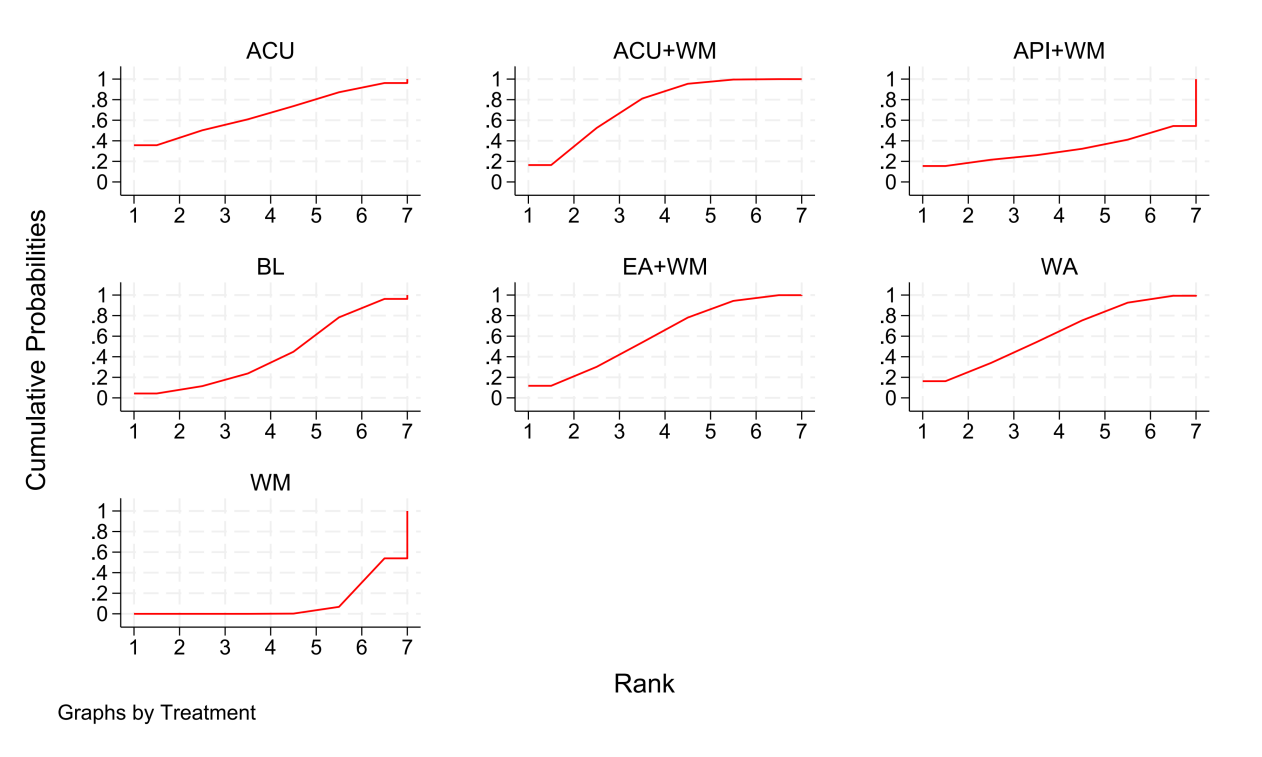


**Table S7.5:** SUCRA of the effects of different acupuncture techniques on Adverse events.

| **Treatment** | **SUCRA** | **PrBest** | **MeanRank** |
| --- | --- | --- | --- |
| ACU | 68.1 | 35.9 | 2.9 |
| ACU+WM | 73.8 | 16.6 | 2.6 |
| API+WM | 30.4 | 14.7 | 5.2 |
| BL | 43.9 | 5.1 | 4.4 |
| EA+WM | 61.1 | 11.8 | 3.3 |
| WA | 62.3 | 16 | 3.3 |
| WM | 10.4 | 0 | 6.4 |

Abbreviations: SUCRA, surface under the cumulative ranking curve.

**Appendix 8:** Pooled efficacy estimates of different acupuncture therapies for primary trigeminal neuralgia from a network meta-analysis of 58 trials

**Table S8.1:** Total Effective Rate

In the league table, the results of comparisons between different acupuncture techniques are displayed in the cells corresponding to each row–column intersection. Effect estimates are reported as mean differences (RR) with 95% confidence intervals (CI).

| ACU |  |  |  |  |  |  |  |  |  |
| --- | --- | --- | --- | --- | --- | --- | --- | --- | --- |
| 1.05 (0.87,1.26) | ACU+WM |  |  |  |  |  |  |  |  |
| 1.06 (0.86,1.30) | 1.01 (0.92,1.12) | API+WM |  |  |  |  |  |  |  |
| 0.62 (0.35,1.11) | 0.59 (0.34,1.03) | 0.59 (0.33,1.03) | BL |  |  |  |  |  |  |
| 1.06 (0.81,1.40) | 1.02 (0.82,1.26) | 1.00 (0.80,1.27) | 1.71 (0.95,3.09) | CAT |  |  |  |  |  |
| 0.90 (0.69,1.17) | 0.86 (0.71,1.04) | 0.85 (0.68,1.05) | 1.45 (0.81,2.59) | 0.84 (0.63,1.12) | EA+WM |  |  |  |  |
| 1.12 (0.82,1.52) | 1.07 (0.83,1.37) | 1.06 (0.81,1.38) | 1.80 (0.98,3.30) | 1.05 (0.76,1.46) | 1.25 (0.91,1.71) | IN+WM |  |  |  |
| 1.10 (0.86,1.40) | 1.05 (0.89,1.24) | 1.04 (0.86,1.25) | 1.77 (0.99,3.14) | 1.03 (0.79,1.34) | 1.22 (0.95,1.57) | 0.98 (0.73,1.32) | WA |  |  |
| 1.03 (0.76,1.39) | 0.98 (0.77,1.25) | 0.97 (0.75,1.26) | 1.66 (0.91,3.03) | 0.97 (0.70,1.33) | 1.15 (0.85,1.56) | 0.92 (0.65,1.30) | 0.94 (0.70,1.26) | WA+WM |  |
| 1.24 (1.04,1.49) | 1.19 (1.15,1.22) | 1.17 (1.07,1.29) | 2.00 (1.15,3.47) | 1.17 (0.95,1.44) | 1.38 (1.14,1.67) | 1.11 (0.87,1.42) | 1.13 (0.96,1.33) | 1.20 (0.95,1.53) | WM |

**Table S8.2:** Visual Analogue Scale

In the league table, the results of comparisons between different acupuncture techniques are displayed in the cells corresponding to each row–column intersection. Effect estimates are reported as mean differences (MD) with 95% confidence intervals (CI).

| ACU+WM |  |  |  |  |  |  |  |  |  |
| --- | --- | --- | --- | --- | --- | --- | --- | --- | --- |
| 1.04 (0.33,3.28) | API+WM |  |  |  |  |  |  |  |  |
| 3.05 (0.32,29.06) | 2.94 (0.25,34.12) | BL |  |  |  |  |  |  |  |
| 0.83 (0.11,6.10) | 0.80 (0.09,7.33) | 0.27 (0.01,5.17) | CAT |  |  |  |  |  |  |
| 0.31 (0.05,1.92) | 0.30 (0.04,2.36) | 0.10 (0.01,1.73) | 0.37 (0.03,5.17) | EA |  |  |  |  |  |
| 0.61 (0.18,2.04) | 0.59 (0.13,2.76) | 0.20 (0.02,2.39) | 0.74 (0.08,6.92) | 1.97 (0.24,15.96) | EA+WM |  |  |  |  |
| 0.68 (0.10,4.86) | 0.66 (0.07,5.86) | 0.22 (0.01,4.15) | 0.82 (0.05,12.53) | 2.19 (0.16,29.69) | 1.11 (0.12,10.24) | IN+WM |  |  |  |
| 0.38 (0.09,1.59) | 0.37 (0.07,2.06) | 0.13 (0.01,1.68) | 0.46 (0.04,4.92) | 1.24 (0.13,11.43) | 0.63 (0.11,3.62) | 0.56 (0.05,5.87) | WA |  |  |
| 0.92 (0.14,6.10) | 0.89 (0.11,7.41) | 0.30 (0.02,5.34) | 1.11 (0.08,16.04) | 2.96 (0.23,37.93) | 1.50 (0.17,12.96) | 1.35 (0.09,19.21) | 2.39 (0.24,23.38) | WA+WM |  |
| 0.20 (0.13,0.32) | 0.20 (0.07,0.57) | 0.07 (0.01,0.61) | 0.25 (0.04,1.72) | 0.66 (0.11,3.87) | 0.34 (0.11,1.03) | 0.30 (0.04,2.04) | 0.53 (0.14,2.05) | 0.22 (0.04,1.40) | WM |

**Table S8.3:** Attack frequency

In the league table, the results of comparisons between different acupuncture techniques are displayed in the cells corresponding to each row–column intersection. Effect estimates are reported as mean differences (MD) with 95% confidence intervals (CI).

|  |  |  |
| --- | --- | --- |
| ACU+WM |  |  |
| 0.00 (0.00,0.10) | EA+WM |  |
| 0.05 (0.02,0.14) | 10.38 (0.59,181.70) | WM |

**Table S8.4:** Traditional Chinese Medicine Syndrome Scoring

In the league table, the results of comparisons between different acupuncture techniques are displayed in the cells corresponding to each row–column intersection. Effect estimates are reported as mean differences (SMD) with 95% confidence intervals (CI).

| ACU+WM |  |  |  |  |  |
| --- | --- | --- | --- | --- | --- |
| 0.22 (0.02,2.01) | API+WM |  |  |  |  |
| 0.06 (0.01,0.60) | 0.30 (0.02,5.72) | CAT |  |  |  |
| 0.24 (0.03,2.22) | 1.11 (0.06,21.04) | 3.69 (0.19,70.48) | EA+WM |  |  |
| 0.49 (0.05,4.57) | 2.27 (0.12,43.24) | 7.59 (0.40,144.83) | 2.05 (0.11,39.10) | WA+WM |  |
| 0.15 (0.07,0.34) | 0.70 (0.09,5.64) | 2.35 (0.29,18.92) | 0.64 (0.08,5.10) | 0.31 (0.04,2.48) | WM |

**Table S8.5:** Adverse events

In the league table, the results of comparisons between different acupuncture techniques are displayed in the cells corresponding to each row–column intersection. Effect estimates are reported as mean differences (OR) with 95% confidence intervals (CI).

| ACU |  |  |  |  |  |  |
| --- | --- | --- | --- | --- | --- | --- |
| 0.99 (0.11,9.20) | ACU+WM |  |  |  |  |  |
| 0.21 (0.00,18.59) | 0.21 (0.00,10.91) | API+WM |  |  |  |  |
| 0.48 (0.04,6.10) | 0.49 (0.13,1.88) | 2.36 (0.04,148.39) | BL |  |  |  |
| 0.77 (0.07,8.62) | 0.78 (0.26,2.35) | 3.77 (0.06,220.25) | 1.60 (0.31,8.19) | EA+WM |  |  |
| 0.78 (0.06,9.92) | 0.79 (0.21,3.06) | 3.83 (0.06,241.22) | 1.62 (0.27,9.94) | 1.02 (0.20,5.21) | WA |  |
| 0.18 (0.02,1.62) | 0.18 (0.12,0.28) | 0.88 (0.02,45.43) | 0.37 (0.10,1.35) | 0.23 (0.09,0.65) | 0.23 (0.06,0.83) | WM |

**Appendix 9: CINeMA Assessment**

We use the CINeMA framework to evidence certainty, assessing it for each network estimate based on the following criteria:

**Within study bias:** We classified the overall risk of bias for each study as low risk of bias, the risk of bias as moderate when none of the four assessed risk of bias items were rated as high risk, and the risk of bias as high when one or both items were rated as high risk. See Appendix 4 for the bias assessment.

**Figure S9.1:** Risk of bias contribution by intervention group in Total Effective Rate

**
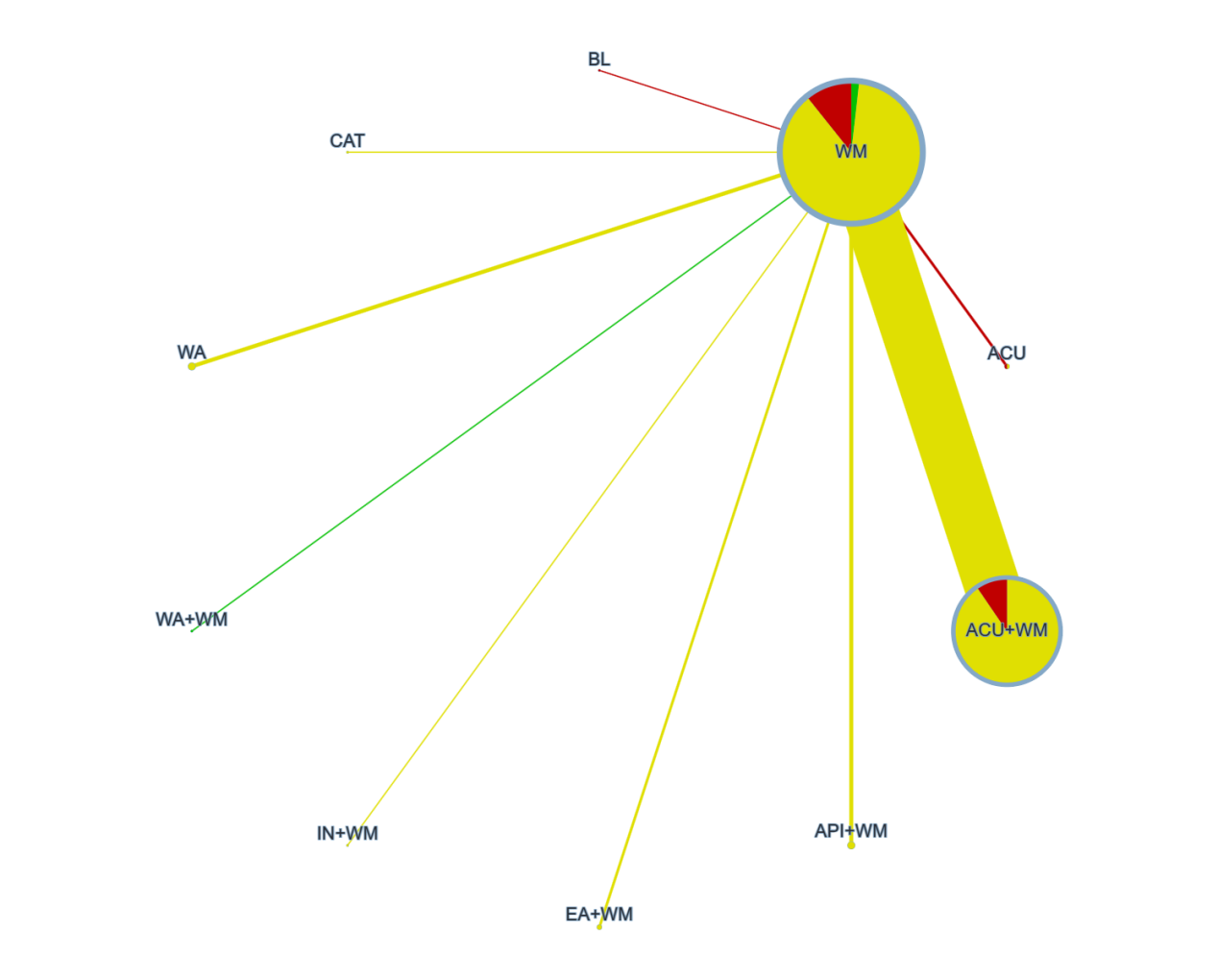
**

**Figure S9.2:** Overall risk of bias by treatment comparison in Total Effective Rate


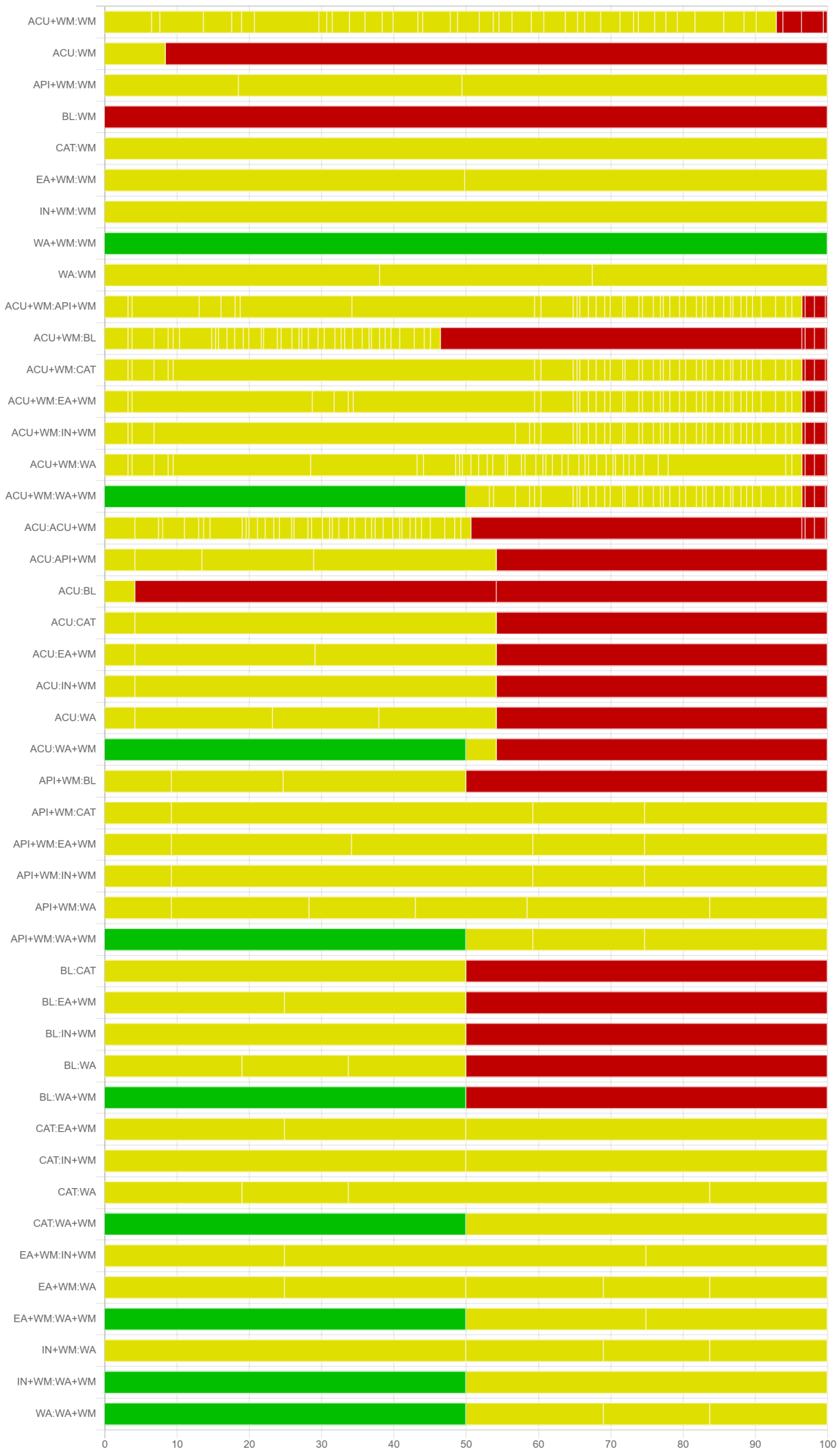


**Figure S9.3:** Risk of bias contribution by intervention group in Visual Analogue Scale


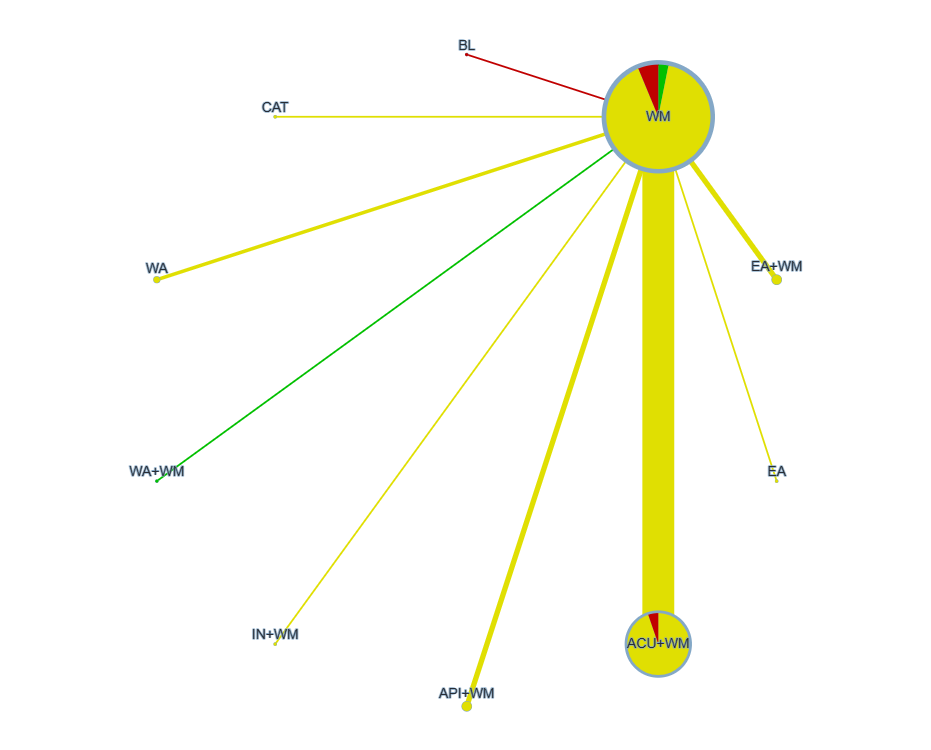


**Figure S9.4:** Overall risk of bias by treatment comparison in Visual Analogue Scale


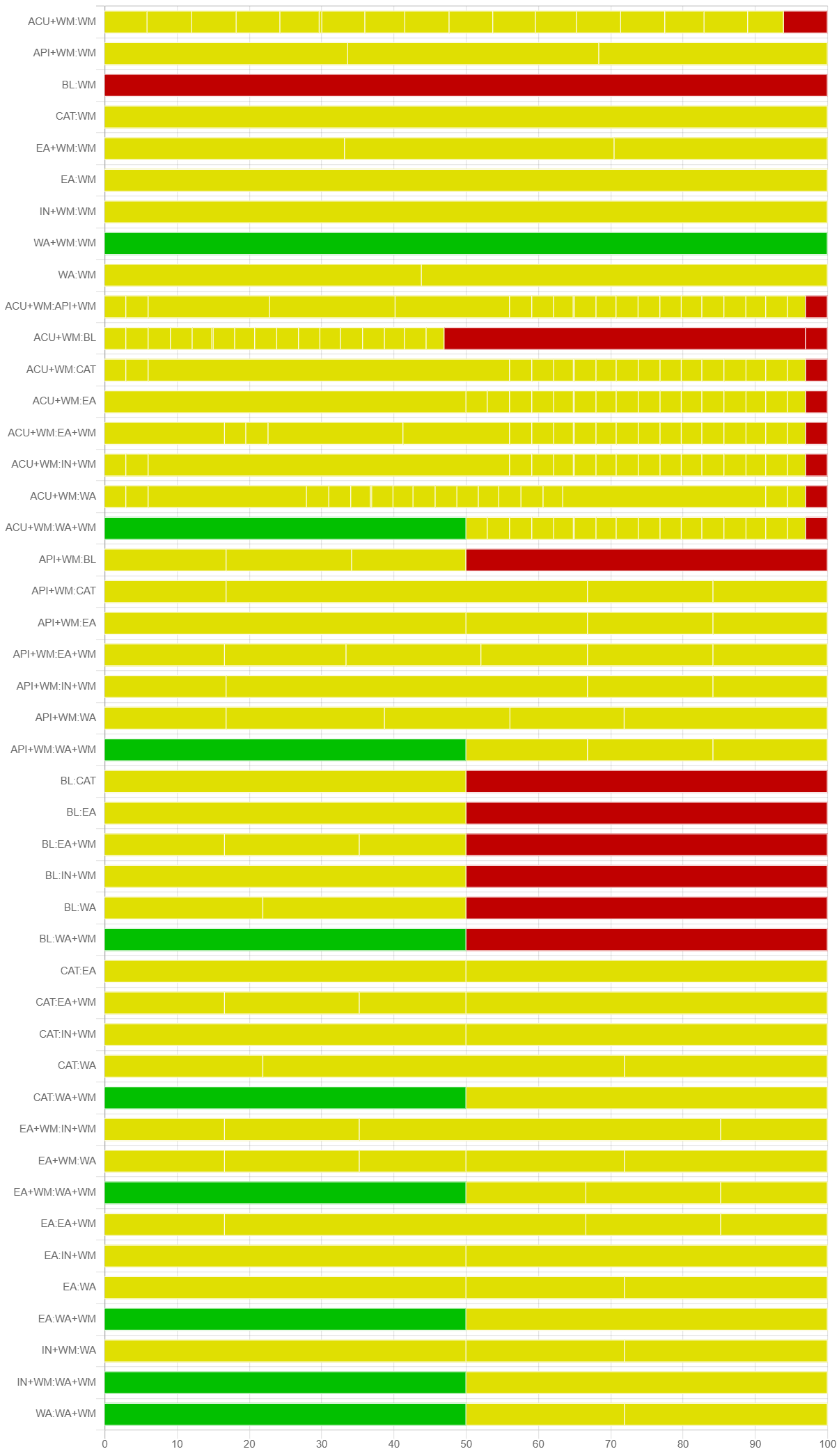


**Figure S9.5:** Risk of bias contribution by intervention group in Attack frequency


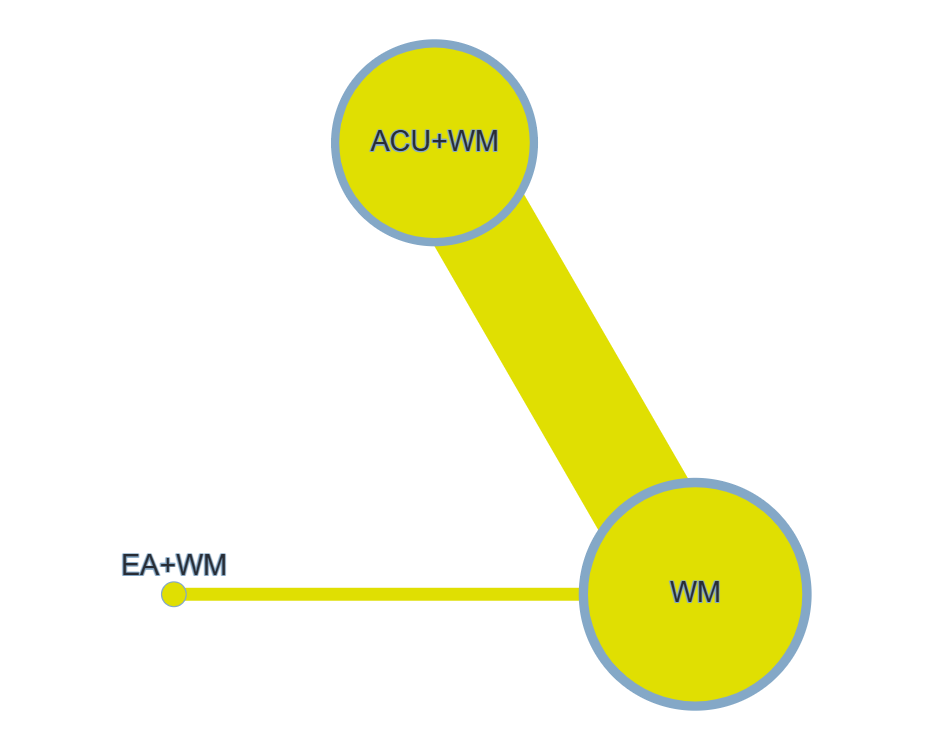


**Figure S9.6:** Overall risk of bias by treatment comparison in Attack frequency


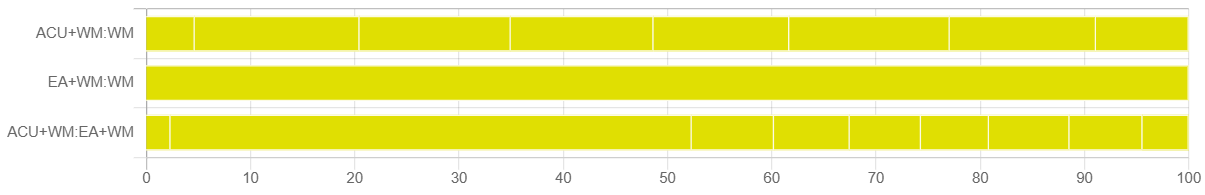


**Figure S9.7:** Risk of bias contribution by intervention group in Traditional Chinese Medicine Syndrome Scoring

**
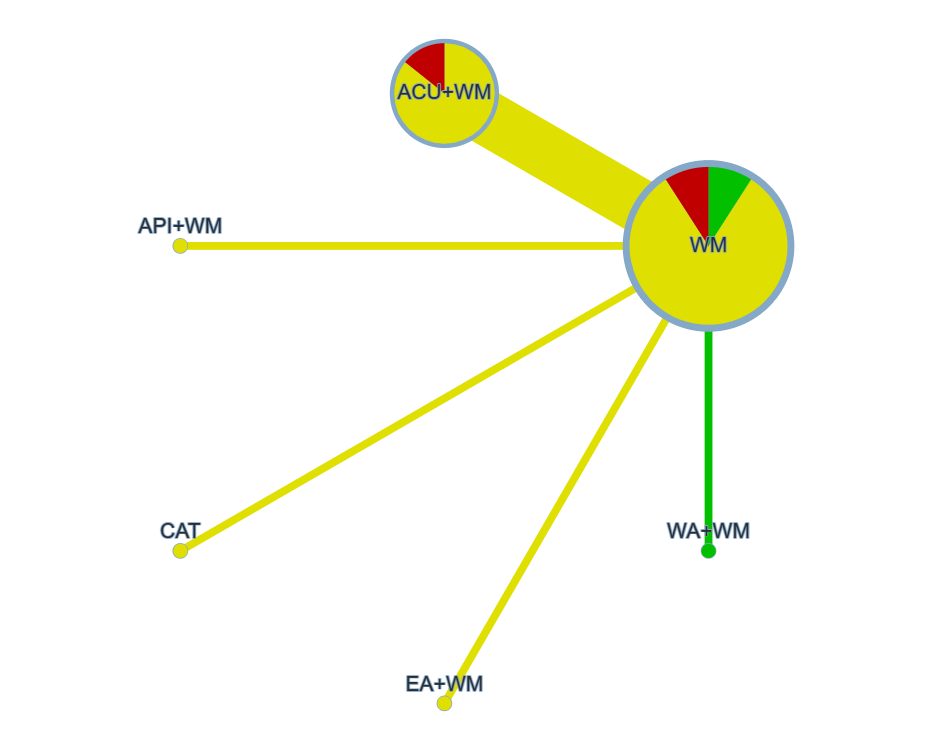
**

**Figure S9.8:** Overall risk of bias by treatment comparison in Traditional Chinese Medicine Syndrome Scoring


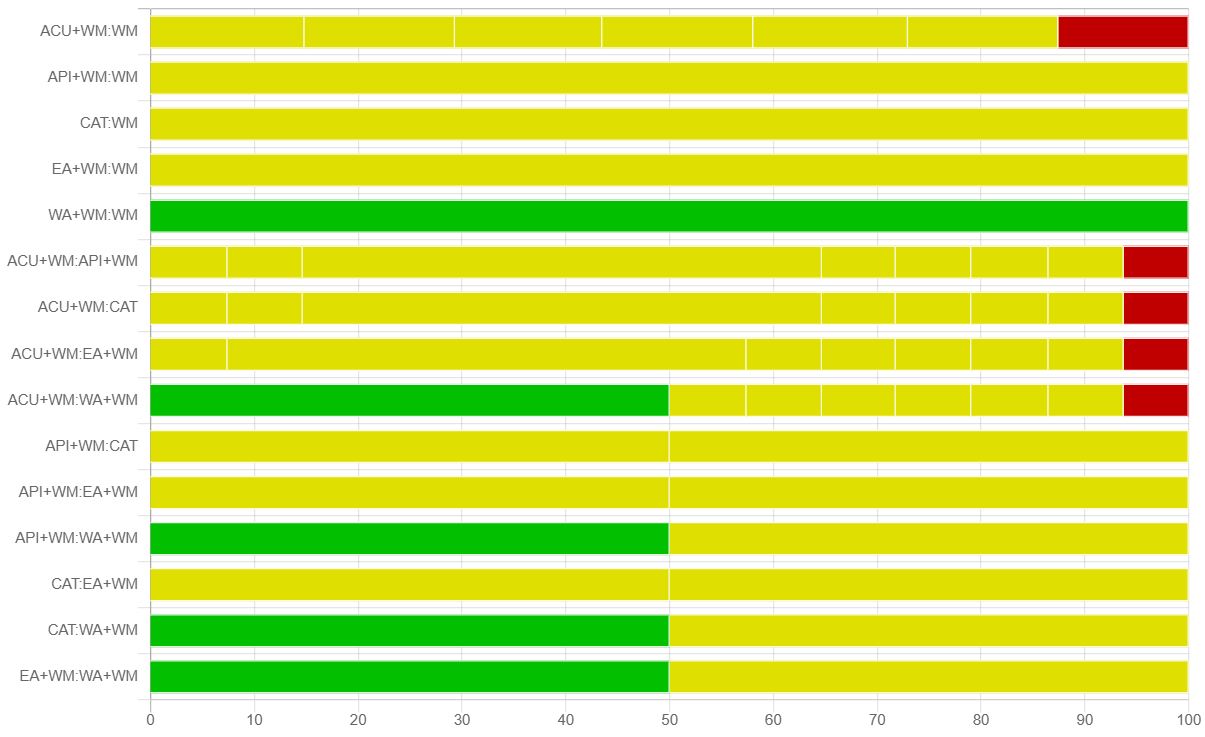


**Figure S9.9:** Risk of bias contribution by intervention group in Adverse events


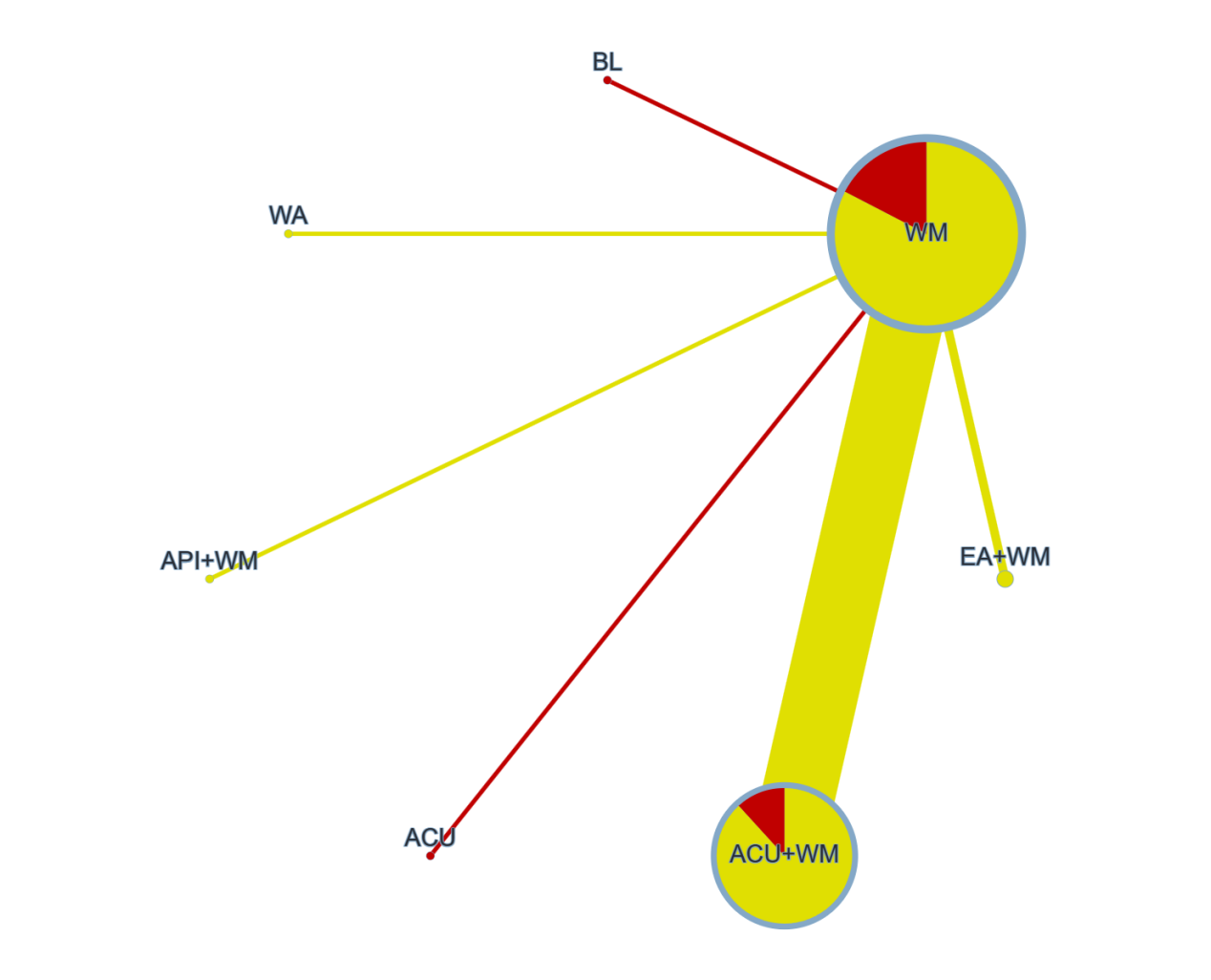


**Figure S9.10:** Overall risk of bias by treatment comparison in Adverse events

**
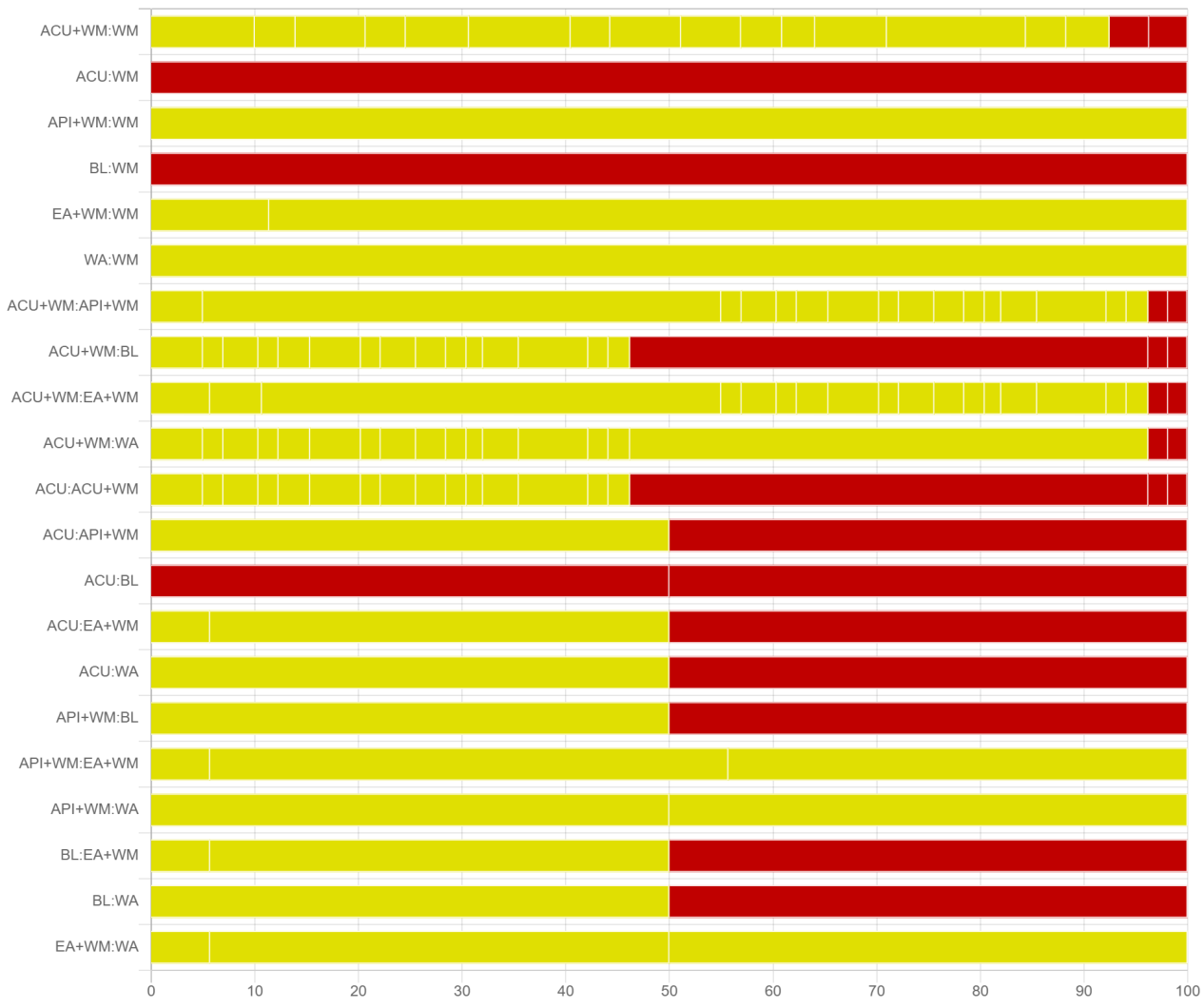
**

**Table S9.1:** Transitivity (Indirectness) Assessment

|  | **Baseline variable (Mean ± SD)** | |
| --- | --- | --- |
| **Intervention** | **Age(year)** | **Course of disease(year)** |
| ACU | 49.19±6.35 | 2.9±1.75 |
| EA | 54.32±7.25 | 1.67±0.82 |
| WA | 47.47±6.64 | 5.17±2.26 |
| CAT | 51.03±8.41 | 16.17±7.69 |
| BL | 63.00±3.50 | 6.63 ± 2.81 |
| ACU+WM | 49.71±6.55 | 4.89 ± 4.94 |
| EA+WM | 53.93±8.24 | 2.36±0.51 |
| API+WM | 48.11±9.29 | 3.87±1.41 |
| IN+WM | 55.00±7.50 | 3.00±1.00 |
| WA+WM | 47.59±7.28 | 2.11±0.52 |

**Imprecision:** We use the CINeMA website to grade the accuracy of each comparison.

**Heterogeneity:** We assessed the degree of worry by comparing clinical reasoning based on 95% confidence intervals (CIs) while applying the same clinical reasoning framework as for inaccuracy.

**Inconsistency:** For inconsistency, we looked at the results for node splitting (Appendix 5) .

**Table S9.2:** CINeMA Results of Total Effective Rate

| **Comparison** | **Within-study bias** | **Reporting bias** | **Indirectness** | **Imprecision** | **Heterogeneity** | **Incoherence** | **Confidence rating** |
| --- | --- | --- | --- | --- | --- | --- | --- |
| ACU:WM | Major concerns | Low risk | No concerns | No concerns | No concerns | No concerns | Low |
| ACU+WM:WM | Some concerns | Low risk | No concerns | No concerns | No concerns | No concerns | Moderate |
| API+WM:WM | Some concerns | Low risk | No concerns | No concerns | No concerns | No concerns | Moderate |
| BL:WM | Major concerns | Low risk | No concerns | No concerns | No concerns | No concerns | Low |
| CAT:WM | Some concerns | Low risk | No concerns | Major concerns | No concerns | No concerns | Very low |
| EA+WM:WM | Some concerns | Low risk | No concerns | No concerns | No concerns | No concerns | Moderate |
| IN+WM:WM | Some concerns | Low risk | No concerns | Major concerns | No concerns | No concerns | Very low |
| WA:WM | Some concerns | Low risk | No concerns | Major concerns | No concerns | No concerns | Very low |
| WA+WM:WM | No concerns | Low risk | No concerns | Major concerns | No concerns | No concerns | Low |
| ACU:ACU+WM | Some concerns | Low risk | No concerns | Major concerns | No concerns | No concerns | Very low |
| ACU:API+WM | Some concerns | Low risk | No concerns | Major concerns | No concerns | No concerns | Very low |
| ACU:BL | Major concerns | Low risk | No concerns | Major concerns | No concerns | No concerns | Very low |
| ACU:CAT | Some concerns | Low risk | No concerns | Major concerns | No concerns | No concerns | Very low |
| ACU:EA+WM | Some concerns | Low risk | No concerns | Major concerns | No concerns | No concerns | Very low |
| ACU:IN+WM | Some concerns | Low risk | No concerns | Major concerns | No concerns | No concerns | Very low |
| ACU:WA | Some concerns | Low risk | No concerns | Major concerns | No concerns | No concerns | Very low |
| ACU:WA+WM | No concerns | Low risk | No concerns | Major concerns | No concerns | No concerns | Low |
| ACU+WM:API+WM | Some concerns | Low risk | No concerns | Major concerns | No concerns | No concerns | Very low |
| ACU+WM:BL | Major concerns | Low risk | No concerns | Major concerns | No concerns | No concerns | Very low |
| ACU+WM:CAT | Some concerns | Low risk | No concerns | Major concerns | No concerns | No concerns | Very low |
| ACU+WM:EA+WM | Some concerns | Low risk | No concerns | Major concerns | No concerns | No concerns | Very low |
| ACU+WM:IN+WM | Some concerns | Low risk | No concerns | Major concerns | No concerns | No concerns | Very low |
| ACU+WM:WA | Some concerns | Low risk | No concerns | Major concerns | No concerns | No concerns | Very low |
| ACU+WM:WA+WM | No concerns | Low risk | No concerns | Major concerns | No concerns | No concerns | Low |
| API+WM:BL | Some concerns | Low risk | No concerns | Major concerns | No concerns | No concerns | Very low |
| API+WM:CAT | Some concerns | Low risk | No concerns | Major concerns | No concerns | No concerns | Very low |
| API+WM:EA+WM | Some concerns | Low risk | No concerns | Major concerns | No concerns | No concerns | Very low |
| API+WM:IN+WM | Some concerns | Low risk | No concerns | Major concerns | No concerns | No concerns | Very low |
| API+WM:WA | Some concerns | Low risk | No concerns | Major concerns | No concerns | No concerns | Very low |
| API+WM:WA+WM | No concerns | Low risk | No concerns | Major concerns | No concerns | No concerns | Low |
| BL:CAT | Some concerns | Low risk | No concerns | Major concerns | No concerns | No concerns | Very low |
| BL:EA+WM | Some concerns | Low risk | No concerns | Major concerns | No concerns | No concerns | Very low |
| BL:IN+WM | Some concerns | Low risk | No concerns | Major concerns | No concerns | No concerns | Very low |
| BL:WA | Some concerns | Low risk | No concerns | Major concerns | No concerns | No concerns | Very low |
| BL:WA+WM | No concerns | Low risk | No concerns | Major concerns | No concerns | No concerns | Low |
| CAT:EA+WM | Some concerns | Low risk | No concerns | Major concerns | No concerns | No concerns | Very low |
| CAT:IN+WM | Some concerns | Low risk | No concerns | Major concerns | No concerns | No concerns | Very low |
| CAT:WA | Some concerns | Low risk | No concerns | Major concerns | No concerns | No concerns | Very low |
| CAT:WA+WM | No concerns | Low risk | No concerns | Major concerns | No concerns | No concerns | Low |
| EA+WM:IN+WM | Some concerns | Low risk | No concerns | Major concerns | No concerns | No concerns | Very low |
| EA+WM:WA | Some concerns | Low risk | No concerns | Major concerns | No concerns | No concerns | Very low |
| EA+WM:WA+WM | No concerns | Low risk | No concerns | Major concerns | No concerns | No concerns | Low |
| IN+WM:WA | Some concerns | Low risk | No concerns | Major concerns | No concerns | No concerns | Very low |
| IN+WM:WA+WM | No concerns | Low risk | No concerns | Major concerns | No concerns | No concerns | Low |
| WA:WA+WM | No concerns | Low risk | No concerns | Major concerns | No concerns | No concerns | Low |

**Table S9.3:** CINeMA Results of Visual Analogue Scale

| **Comparison** | **Within-study bias** | **Reporting bias** | **Indirectness** | **Imprecision** | **Heterogeneity** | **Incoherence** | **Confidence rating** |
| --- | --- | --- | --- | --- | --- | --- | --- |
| ACU+WM:WM | Some concerns | Low risk | No concerns | No concerns | Major concerns | Major concerns | Very low |
| API+WM:WM | Some concerns | Low risk | No concerns | No concerns | Major concerns | Major concerns | Very low |
| BL:WM | Major concerns | Low risk | No concerns | No concerns | Major concerns | Major concerns | Very low |
| CAT:WM | Some concerns | Low risk | No concerns | Major concerns | No concerns | Major concerns | Very low |
| EA:WM | Some concerns | Low risk | No concerns | Major concerns | No concerns | Major concerns | Very low |
| EA+WM:WM | Some concerns | Low risk | No concerns | Major concerns | No concerns | Major concerns | Very low |
| IN+WM:WM | Some concerns | Low risk | No concerns | Major concerns | No concerns | Major concerns | Very low |
| WA:WM | Some concerns | Low risk | No concerns | Major concerns | No concerns | Major concerns | Very low |
| WA+WM:WM | No concerns | Low risk | No concerns | Major concerns | No concerns | Major concerns | Very low |
| ACU+WM:API+WM | Some concerns | Low risk | No concerns | Major concerns | No concerns | Major concerns | Very low |
| ACU+WM:BL | Major concerns | Low risk | No concerns | Major concerns | No concerns | Major concerns | Very low |
| ACU+WM:CAT | Some concerns | Low risk | No concerns | Major concerns | No concerns | Major concerns | Very low |
| ACU+WM:EA | Some concerns | Low risk | No concerns | Major concerns | No concerns | Major concerns | Very low |
| ACU+WM:EA+WM | Some concerns | Low risk | No concerns | Major concerns | No concerns | Major concerns | Very low |
| ACU+WM:IN+WM | Some concerns | Low risk | No concerns | Major concerns | No concerns | Major concerns | Very low |
| ACU+WM:WA | Some concerns | Low risk | No concerns | Major concerns | No concerns | Major concerns | Very low |
| ACU+WM:WA+WM | No concerns | Low risk | No concerns | Major concerns | No concerns | Major concerns | Very low |
| API+WM:BL | Major concerns | Low risk | No concerns | Major concerns | No concerns | Major concerns | Very low |
| API+WM:CAT | Some concerns | Low risk | No concerns | Major concerns | No concerns | Major concerns | Very low |
| API+WM:EA | Some concerns | Low risk | No concerns | Major concerns | No concerns | Major concerns | Very low |
| API+WM:EA+WM | Some concerns | Low risk | No concerns | Major concerns | No concerns | Major concerns | Very low |
| API+WM:IN+WM | Some concerns | Low risk | No concerns | Major concerns | No concerns | Major concerns | Very low |
| API+WM:WA | Some concerns | Low risk | No concerns | Major concerns | No concerns | Major concerns | Very low |
| API+WM:WA+WM | No concerns | Low risk | No concerns | Major concerns | No concerns | Major concerns | Very low |
| BL:CAT | Some concerns | Low risk | No concerns | Major concerns | No concerns | Major concerns | Very low |
| BL:EA | Some concerns | Low risk | No concerns | Major concerns | No concerns | Major concerns | Very low |
| BL:EA+WM | Some concerns | Low risk | No concerns | Major concerns | No concerns | Major concerns | Very low |
| BL:IN+WM | Some concerns | Low risk | No concerns | Major concerns | No concerns | Major concerns | Very low |
| BL:WA | Some concerns | Low risk | No concerns | Major concerns | No concerns | Major concerns | Very low |
| BL:WA+WM | No concerns | Low risk | No concerns | Major concerns | No concerns | Major concerns | Very low |
| CAT:EA | Some concerns | Low risk | No concerns | Major concerns | No concerns | Major concerns | Very low |
| CAT:EA+WM | Some concerns | Low risk | No concerns | Major concerns | No concerns | Major concerns | Very low |
| CAT:IN+WM | Some concerns | Low risk | No concerns | Major concerns | No concerns | Major concerns | Very low |
| CAT:WA | Some concerns | Low risk | No concerns | Major concerns | No concerns | Major concerns | Very low |
| CAT:WA+WM | No concerns | Low risk | No concerns | Major concerns | No concerns | Major concerns | Very low |
| EA:EA+WM | Some concerns | Low risk | No concerns | Major concerns | No concerns | Major concerns | Very low |
| EA:IN+WM | Some concerns | Low risk | No concerns | Major concerns | No concerns | Major concerns | Very low |
| EA:WA | Some concerns | Low risk | No concerns | Major concerns | No concerns | Major concerns | Very low |
| EA:WA+WM | No concerns | Low risk | No concerns | Major concerns | No concerns | Major concerns | Very low |
| EA+WM:IN+WM | Some concerns | Low risk | No concerns | Major concerns | No concerns | Major concerns | Very low |
| EA+WM:WA | Some concerns | Low risk | No concerns | Major concerns | No concerns | Major concerns | Very low |
| EA+WM:WA+WM | No concerns | Low risk | No concerns | Major concerns | No concerns | Major concerns | Very low |
| IN+WM:WA | Some concerns | Low risk | No concerns | Major concerns | No concerns | Major concerns | Very low |
| IN+WM:WA+WM | No concerns | Low risk | No concerns | Major concerns | No concerns | Major concerns | Very low |
| WA:WA+WM | No concerns | Low risk | No concerns | Major concerns | No concerns | Major concerns | Very low |

**Table S9.4:** CINeMA Results of Attack frequency

| **Comparison** | **Within-study bias** | **Reporting bias** | **Indirectness** | **Imprecision** | **Heterogeneity** | **Incoherence** | **Confidence rating** |
| --- | --- | --- | --- | --- | --- | --- | --- |
| ACU+WM:WM | Some concerns | Low risk | No concerns | No concerns | Major concerns | Major concerns | Very low |
| EA+WM:WM | Some concerns | Low risk | No concerns | Major concerns | No concerns | Major concerns | Very low |
| ACU+WM:EA+WM | Some concerns | Low risk | No concerns | No concerns | Major concerns | Major concerns | Very low |

**Table S9.5:** CINeMA Results of Traditional Chinese Medicine Syndrome Scoring

| **Comparison** | **Within-study bias** | **Reporting bias** | **Indirectness** | **Imprecision** | **Heterogeneity** | **Incoherence** | **Confidence rating** |
| --- | --- | --- | --- | --- | --- | --- | --- |
| ACU+WM:WM | Some concerns | Low risk | No concerns | No concerns | Major concerns | Major concerns | Very low |
| API+WM:WM | Some concerns | Low risk | No concerns | Major concerns | No concerns | Major concerns | Very low |
| CAT:WM | Some concerns | Low risk | No concerns | Major concerns | No concerns | Major concerns | Very low |
| EA+WM:WM | Some concerns | Low risk | No concerns | Major concerns | No concerns | Major concerns | Very low |
| WA+WM:WM | No concerns | Low risk | No concerns | Major concerns | No concerns | Major concerns | Very low |
| ACU+WM:API+WM | Some concerns | Low risk | No concerns | Major concerns | No concerns | Major concerns | Very low |
| ACU+WM:CAT | Some concerns | Low risk | No concerns | No concerns | Major concerns | Major concerns | Very low |
| ACU+WM:EA+WM | Some concerns | Low risk | No concerns | Major concerns | No concerns | Major concerns | Very low |
| ACU+WM:WA+WM | No concerns | Low risk | No concerns | Major concerns | No concerns | Major concerns | Very low |
| API+WM:CAT | Some concerns | Low risk | No concerns | Major concerns | No concerns | Major concerns | Very low |
| API+WM:EA+WM | Some concerns | Low risk | No concerns | Major concerns | No concerns | Major concerns | Very low |
| API+WM:WA+WM | No concerns | Low risk | No concerns | Major concerns | No concerns | Major concerns | Very low |
| CAT:EA+WM | Some concerns | Low risk | No concerns | Major concerns | No concerns | Major concerns | Very low |
| CAT:WA+WM | No concerns | Low risk | No concerns | Major concerns | No concerns | Major concerns | Very low |
| EA+WM:WA+WM | No concerns | Low risk | No concerns | Major concerns | No concerns | Major concerns | Very low |

**Table S9.6:** CINeMA Results of Adverse events

| **Comparison** | **Within-study bias** | **Reporting bias** | **Indirectness** | **Imprecision** | **Heterogeneity** | **Incoherence** | **Confidence rating** |
| --- | --- | --- | --- | --- | --- | --- | --- |
| ACU:WM | Major concerns | Low risk | No concerns | Major concerns | No concerns | No concerns | Very low |
| ACU+WM:WM | Some concerns | Low risk | No concerns | No concerns | No concerns | No concerns | Moderate |
| API+WM:WM | Some concerns | Low risk | No concerns | Major concerns | No concerns | No concerns | Very low |
| BL:WM | Major concerns | Low risk | No concerns | Major concerns | No concerns | No concerns | Very low |
| EA+WM:WM | Some concerns | Low risk | No concerns | No concerns | No concerns | No concerns | Moderate |
| WA:WM | Some concerns | Low risk | No concerns | No concerns | No concerns | No concerns | Moderate |
| ACU:ACU+WM | Major concerns | Low risk | No concerns | Major concerns | No concerns | No concerns | Very low |
| ACU:API+WM | Some concerns | Low risk | No concerns | Major concerns | No concerns | No concerns | Very low |
| ACU:BL | Major concerns | Low risk | No concerns | Major concerns | No concerns | No concerns | Very low |
| ACU:EA+WM | Some concerns | Low risk | No concerns | No concerns | No concerns | No concerns | Moderate |
| ACU:WA | Some concerns | Low risk | No concerns | No concerns | No concerns | No concerns | Moderate |
| ACU+WM:API+WM | Some concerns | Low risk | No concerns | Major concerns | No concerns | No concerns | Very low |
| ACU+WM:BL | Major concerns | Low risk | No concerns | Major concerns | No concerns | No concerns | Very low |
| ACU+WM:EA+WM | Some concerns | Low risk | No concerns | No concerns | No concerns | No concerns | Moderate |
| ACU+WM:WA | Some concerns | Low risk | No concerns | No concerns | No concerns | No concerns | Moderate |
| API+WM:BL | Some concerns | Low risk | No concerns | Major concerns | No concerns | No concerns | Very low |
| API+WM:EA+WM | Some concerns | Low risk | No concerns | Major concerns | No concerns | No concerns | Very low |
| API+WM:WA | Some concerns | Low risk | No concerns | Major concerns | No concerns | No concerns | Very low |
| BL:EA+WM | Some concerns | Low risk | No concerns | No concerns | No concerns | No concerns | Moderate |
| BL:WA | Some concerns | Low risk | No concerns | No concerns | No concerns | No concerns | Moderate |
| EA+WM:WA | Some concerns | Low risk | No concerns | Major concerns | No concerns | No concerns | Very low |

**Appendix 10: Funnel plots**

The figure shows the evaluation of small-study effects, indicative of potential publication bias, among studies examining the effects of different acupuncture techniques on various outcomes in primary trigeminal neuralgia.

**Figure S10.1:** Total Effective Rate


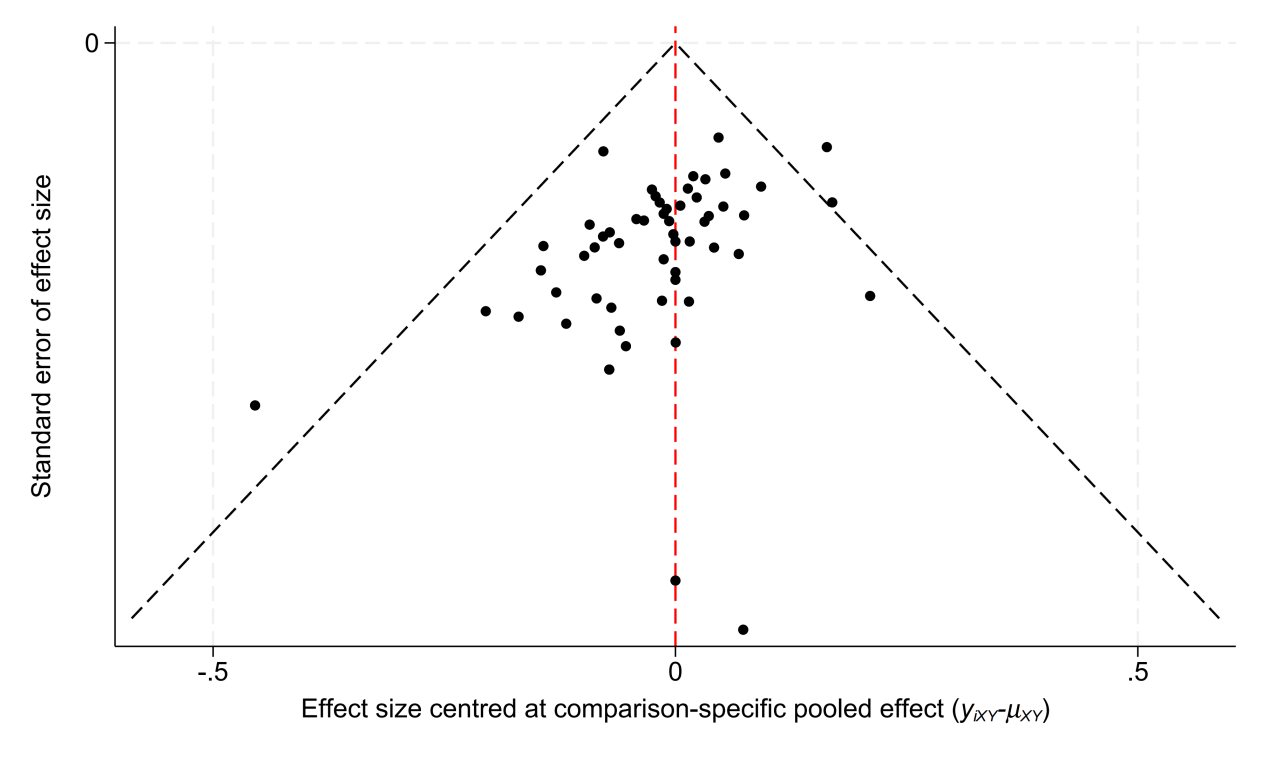


**Figure S10.2:** Visual Analogue Scale


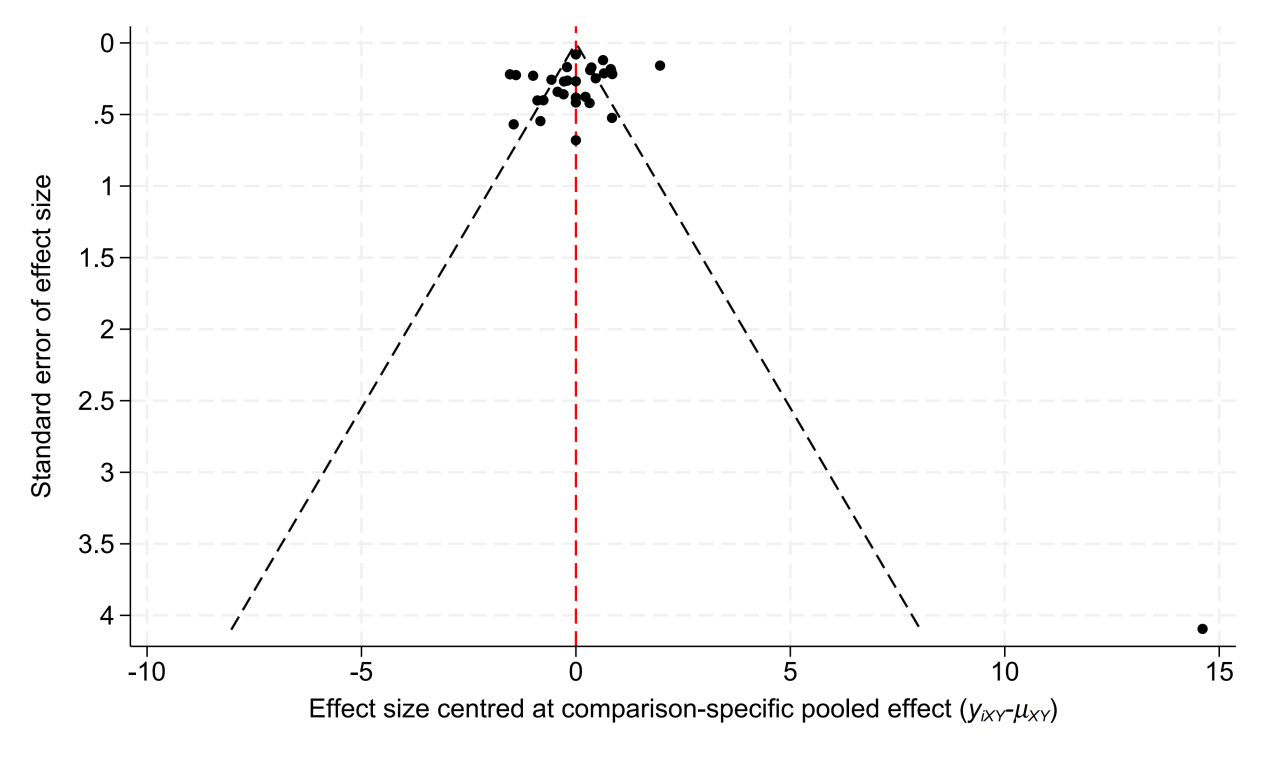


**Figure S10.3:** Attack frequency


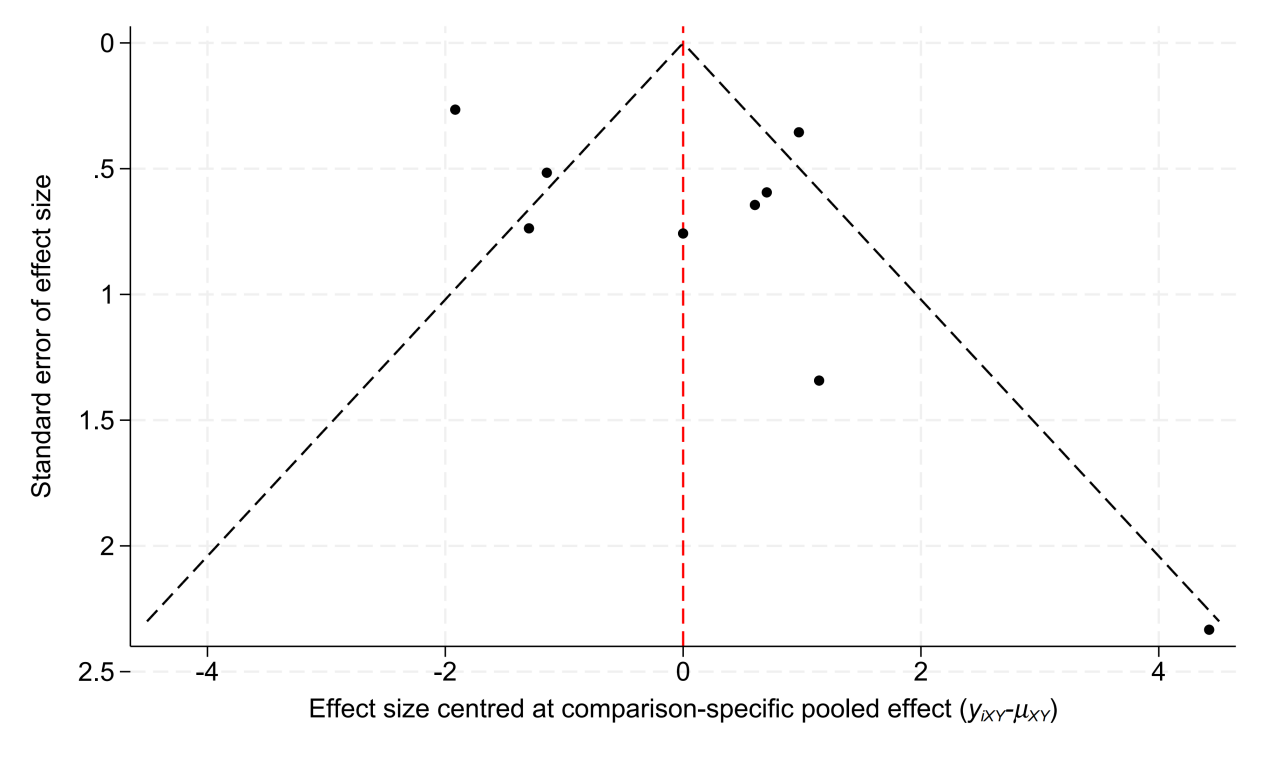


**Figure S10.4:** Traditional Chinese Medicine Syndrome Scoring


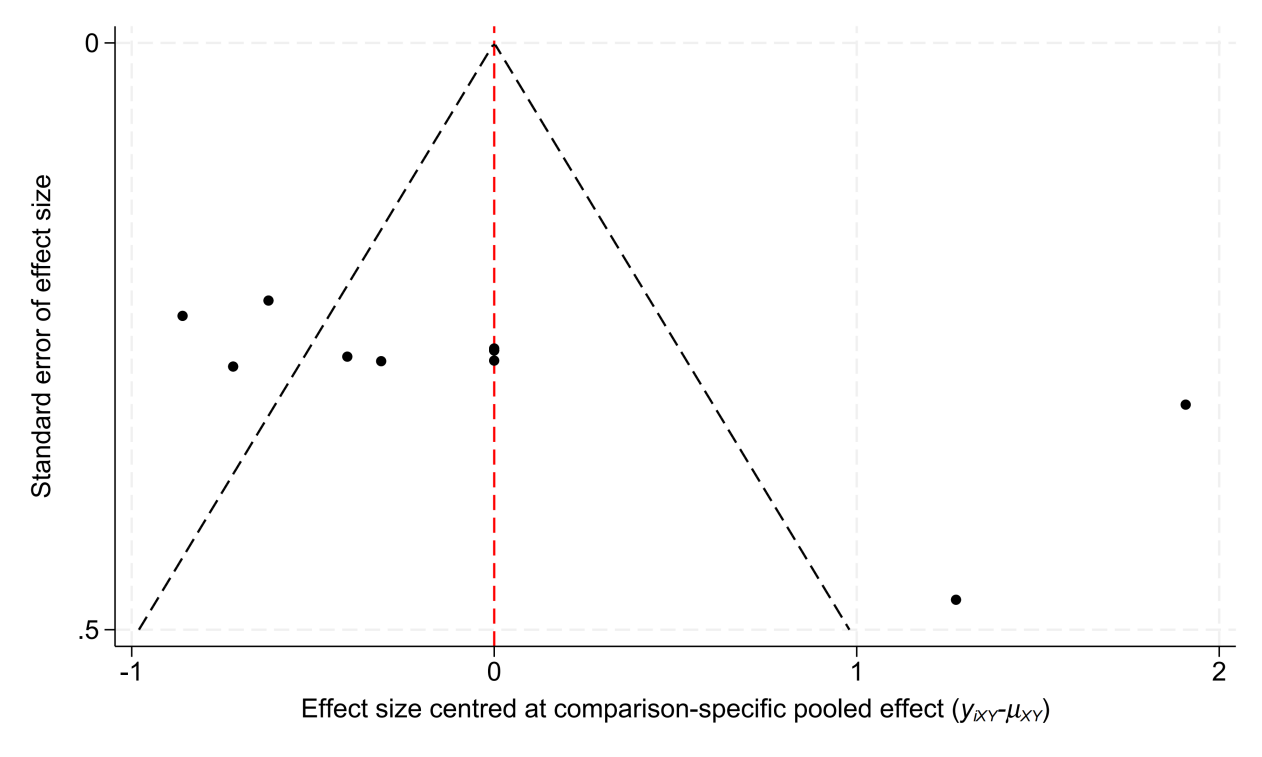


**Figure S10.5:** Adverse events

**
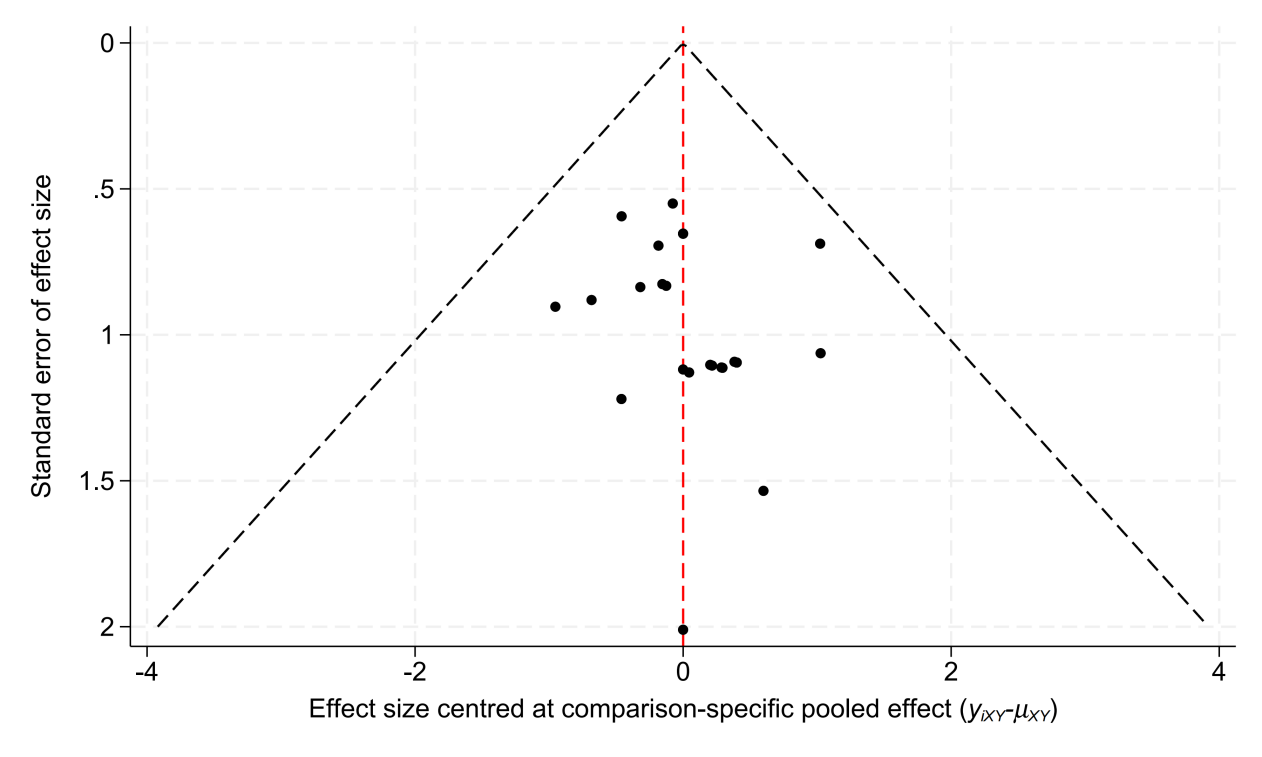
**

**Appendix 11: Sensitivity analyses**

**Figure S11.1:** Sensitivity analyses of Excluding Studies at High Risk of Bias

| **Different acupuncture therapies** | **Total Effective Rate** | | **Visual Analogue Scale** | | **Attack frequency** | | **Traditional Chinese Medicine Syndrome Scoring** | | **Adverse events** | |
| --- | --- | --- | --- | --- | --- | --- | --- | --- | --- | --- |
|  | **Main estimate** | **Sensitivity analyses** | **Main estimate** | **Sensitivity analyses** | **Main estimate** | **Sensitivity analyses** | **Main estimate** | **Sensitivity analyses** | **Main estimate** | **Sensitivity analyses** |
| ACU | 1.24 (1.04,1.49) | 0.98 (0.54,1.77) | NA | NA | NA | NA | NA | NA | 0.18 (0.02,1.62) | NA |
| ACU+WM | 1.19 (1.15,1.22) | 0.98 (0.54,1.79) | 0.20 (0.13,0.32) | 0.21 (0.13,0.34) | 0.05 (0.02,0.14) | 0.05 (0.02,0.14) | 0.15 (0.07,0.34) | 0.18 (0.08,0.41) | 0.18 (0.12,0.28) | 0.19 (0.12,0.29) |
| API+WM | 1.17 (1.07,1.29) | 0.99 (0.53,1.85) | 0.20 (0.07,0.57) | 0.25 (0.03,1.75) | NA | NA | 0.70 (0.09,5.64) | 0.70 (0.10,5.16) | 0.88 (0.02,45.43) | 0.88 (0.02,45.43) |
| BL | 2.00 (1.15,3.47) | NA | 0.07 (0.01,0.61) | NA | NA | NA | NA | NA | 0.37 (0.10,1.35) | 0.37 (0.10,1.35) |
| CAT | 1.17 (0.95,1.44) | 0.83 (0.45,1.55) | 0.25 (0.04,1.72) | 0.25 (0.03,1.75) | NA | NA | 2.35 (0.29,18.92) | 2.35 (0.32,17.32) | NA | NA |
| EA | NA | NA | 0.66 (0.11,3.87) | 0.66 (0.11,3.95) | NA | NA | NA | NA | NA | NA |
| EA+WM | 1.38 (1.14,1.67) | 1.04 (0.55,1.97) | 0.34 (0.11,1.03) | 0.34 (0.11,1.04) | 10.38 (0.59,181.70) | 10.38 (0.59,181.70) | 0.64 (0.08,5.10) | 0.64 (0.09,4.67) | 0.23 (0.09,0.65) | 0.23 (0.09,0.65) |
| IN+WM | 1.11 (0.87,1.42) | 1.02 (0.55,1.88) | 0.30 (0.04,2.04) | 0.30 (0.04,2.08) | NA | NA | NA | NA | NA | NA |
| WA | 1.13 (0.96,1.33) | 0.96 (0.50,1.81) | 0.53 (0.14,2.05) | 0.53 (0.14,2.09) | NA | NA | NA | NA | 0.23 (0.06,0.83) | 0.23 (0.06,0.83) |
| WA+WM | 1.20 (0.95,1.53) | 1.15 (0.64,2.08) | 0.22 (0.04,1.40) | 0.22 (0.03,1.43) | NA | NA | 0.31 (0.04,2.48) | 0.31 (0.04,2.27) | NA | NA |

**Figure S11.2：**Sensitivity Analysis Excluding Studies Combining Acupuncture With Conventional Western Medicine

| **Different acupuncture therapies** | **Total Effective Rate** | | **Visual Analogue Scale** | |
| --- | --- | --- | --- | --- |
|  | **Main estimate** | **Sensitivity analyses** | **Main estimate** | **Sensitivity analyses** |
| ACU | 1.24 (1.04,1.49) | 1.23 (0.95,1.61) | NA | NA |
| ACU+WM | 1.19 (1.15,1.22) | NA | 0.20 (0.13,0.32) | NA |
| API+WM | 1.17 (1.07,1.29) | NA | 0.20 (0.07,0.57) | NA |
| BL | 2.00 (1.15,3.47) | 2.00 (1.10,3.62) | 0.07 (0.01,0.61) | 0.07 (0.01,0.67) |
| CAT | 1.17 (0.95,1.44) | 1.17 (0.86,1.58) | 0.25 (0.04,1.72) | 0.25 (0.03,1.84) |
| EA | NA | NA | 0.66 (0.11,3.87) | 0.66 (0.10,4.14) |
| EA+WM | 1.38 (1.14,1.67) | NA | 0.34 (0.11,1.03) | NA |
| IN+WM | 1.11 (0.87,1.42) | NA | 0.30 (0.04,2.04) | NA |
| WA | 1.13 (0.96,1.33) | 1.14 (0.92,1.40) | 0.53 (0.14,2.05) | 0.54 (0.13,2.22) |
| WA+WM | 1.20 (0.95,1.53) | NA | 0.22 (0.04,1.40) | NA |

**Appendix 12: Regression analyses**

| **Factor** |  | | |  | | |  | | |  | | |  | | |
| --- | --- | --- | --- | --- | --- | --- | --- | --- | --- | --- | --- | --- | --- | --- | --- |
|  | **Coefficient** | **95% confidence interval** | **P value** | **Coefficient** | **95% confidence interval** | **P value** | **Coefficient** | **95% confidence interval** | **P value** | **Coefficient** | **95% confidence interval** | **P value** | **Coefficient** | **95% confidence interval** | **P value** |
| Treatment duration | 1.00 | 0.88 1.14 | 0.92 | 0.31 | -.63 1.26 | 0.50 | -1.65 | -5.14 1.82 | 0.30 | 0.29 | -2.87 3.45 | 0.84 | 1.21 | 0.57 2.56 | 0.59 |
